# Synthesis, Computational Insights, and Evaluation of Novel Sigma Receptors Ligands

**DOI:** 10.1021/acschemneuro.3c00074

**Published:** 2023-05-08

**Authors:** Maria Dichiara, Francesca Alessandra Ambrosio, Carla Barbaraci, Rafael González-Cano, Giosuè Costa, Carmela Parenti, Agostino Marrazzo, Lorella Pasquinucci, Enrique J. Cobos, Stefano Alcaro, Emanuele Amata

**Affiliations:** † Dipartimento di Scienze del Farmaco e della Salute, Università degli Studi di Catania, Viale Andrea Doria 6, 95125 Catania, Italy; ‡ Dipartimento di Medicina Sperimentale e Clinica, Università degli Studi “Magna Græcia” di Catanzaro, Campus “S. Venuta”, Viale Europa, 88100 Catanzaro, Italy; § Departamento de Farmacología e Instituto de Neurociencias, Facultad de Medicina, Universitad de Granada e Instituto de Investigación Biosanitaria de Granada ibs.GRANADA, Avenida de la Investigación 11, 18016 Granada, Spain; ∥ Dipartimento di Scienze della Salute, Università “Magna Græcia” di Catanzaro, Campus “S. Venuta”, 88100 Catanzaro, Italy; ⊥ Net4Science Academic Spin-Off, Università “Magna Græcia” di Catanzaro, Campus “S. Venuta”, 88100 Catanzaro, Italy

**Keywords:** sigma receptors, SR ligands, S1R agonist, S1R antagonist, drug discovery, molecular modeling, mechanical hypersensitivity

## Abstract

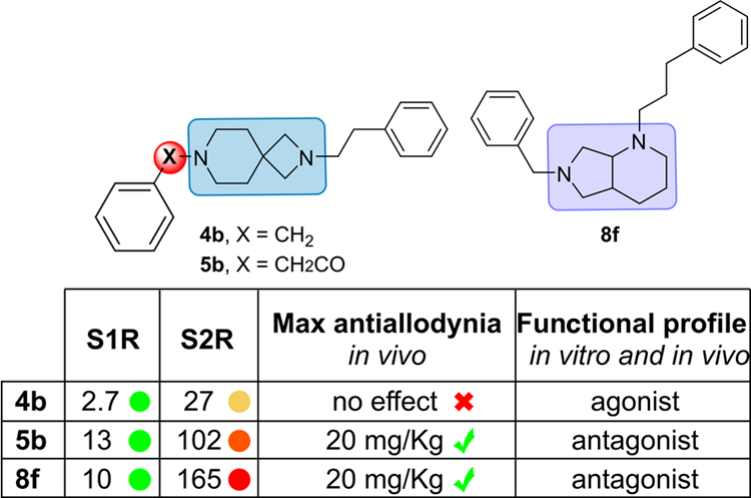

The development of diazabicyclo[4.3.0]nonane and 2,7-diazaspiro[3.5]nonane derivatives as sigma receptors (SRs) ligands is reported. The compounds were evaluated in S1R and S2R binding assays, and modeling studies were carried out to analyze the binding mode. The most notable compounds, **4b** (AD186, *K*_i_S1R = 2.7 nM, *K*_i_S2R = 27 nM), **5b** (AB21, *K*_i_S1R = 13 nM, *K*_i_S2R = 102 nM), and **8f** (AB10, *K*_i_S1R = 10 nM, *K*_i_S2R = 165 nM), have been screened for analgesic effects *in vivo*, and their functional profile was determined through *in vivo* and *in vitro* models. Compounds **5b** and **8f** reached the maximum antiallodynic effect at 20 mg/kg. The selective S1R agonist PRE-084 completely reversed their action, indicating that the effects are entirely dependent on the S1R antagonism. Conversely, compound **4b** sharing the 2,7-diazaspiro[3.5]nonane core as **5b** was completely devoid of antiallodynic effect. Interestingly, compound **4b** fully reversed the antiallodynic effect of BD-1063, indicating that **4b** induces an S1R agonistic *in vivo* effect. The functional profiles were confirmed by the phenytoin assay. Our study might establish the importance of 2,7-diazaspiro[3.5]nonane core for the development of S1R compounds with specific agonist or antagonist profile and the role of the diazabicyclo[4.3.0]nonane in the development of novel SR ligands.

## Introduction

**Figure 1:**
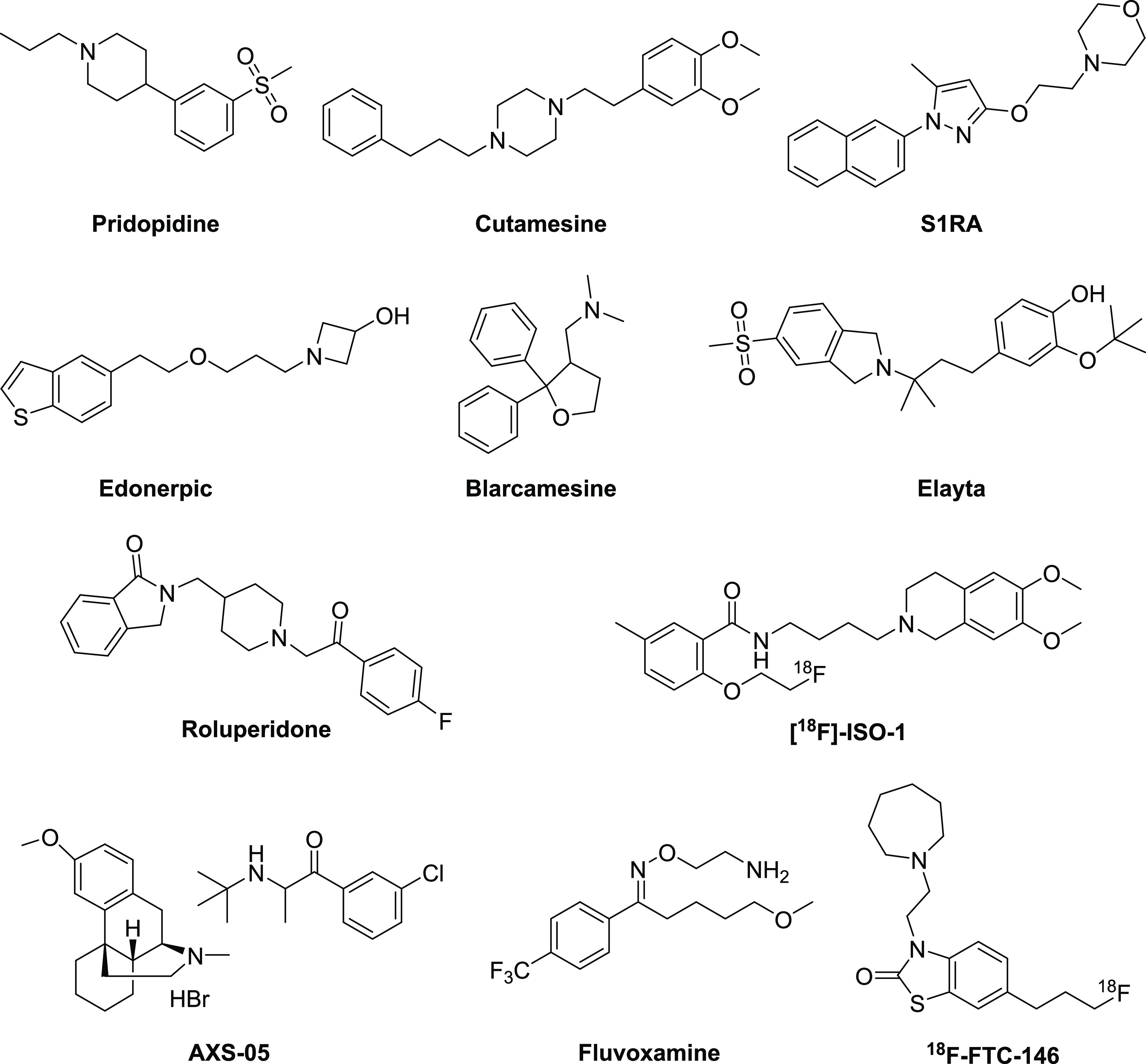
Chemical structures of SR ligands evaluated in clinical trials.

Sigma receptors (SRs)—termed as sigma-1 (S1R) and sigma-2 (S2R) receptor—are involved in several biological and pathological conditions.^1^ S1R is a chaperone protein mainly localized at the mitochondria-associated membrane (MAM) of the endoplasmic reticulum (ER) where it forms a complex with the binding immunoglobulin protein (BiP).^2,3^ Upon activation, S1R dissociates from BiP moving toward the plasma membrane where it directly interacts with different ion channels and G-protein-coupled receptors.^4−6^ The S1R is highly expressed in both central and peripheral nervous systems, in areas of great relevance for neuroprotection and neuroinflammation.^7^ S1R ligands have historically been classified as agonists or antagonists, with agonists favoring oligomerization and antagonists preventing it based on their effects in a variety of *in vivo* or cellular models. Receptor antagonists are reported to have analgesic effects in both animals and humans and to enhance opioid mediated analgesia.^8,9^ On the contrary, S1R agonists oppose the effects of antagonists, and they are associated with cytoprotective effects.^10,11^ So far, five selective S1R ligands have been advanced into later phase clinical studies for the treatment of various human conditions (Figure 1). Blarcamesine (ANAVEX2-73) is an S1R and muscarinic (M1) receptor agonist under phase 2/3 investigation in patients with Alzheimer’s disease, Rett syndrome, and Parkinson’s disease with dementia.^12−14^ Although S1R plays a role in the phosphorylation of Tau protein, with a reduction of S1R expression resulting in an increase in Tau hyperphosphorylation, M1 receptor agonists also attenuate Tau hyperphosphorylation.^15^ Pridopidine (ACR16)—an S1R agonist and dopamine stabilizer with 100-fold greater affinity for S1R than for D2—is aimed at alleviating motor impairments and increasing neuron survival through the alteration dopaminergic transmission and modulation of the S1R. The candidate drug is in phase 3 clinical for the early-stage and symptomatic treatment of Huntington’s disease,^16−18^ and in phase 2 for the treatment of levodopa-induced dyskinesia in Parkinson’s disease patients.^19^ Cutamesine (SA4503) is an S1R agonist with selectivity over 36 other receptors, under phase 2 clinical trial in subjects with major depressive disorder, able to restore motor function after acute ischemic stroke.^20,21^ The selective S1R antagonist E-52862 (S1RA) has been evaluated in a phase II clinical trial for the treatment of neuropathic pain of different etiology. The neurotropic agent Edonerpic (T-817MA) has advanced into phase II clinical trials in patients with mild to moderate Alzheimer’s disease, even if the molecular mechanisms underlying these effects are not fully understood.^22^ The uncompetitive NMDA receptor antagonist and S1R agonist dextromethorphan hydrobromide have been positively evaluated in combination (AXS-05) with the antidepressant bupropion hydrochloride—an aminoketone and CYP2D6 inhibitor that increases dextromethorphan bioavailability—in the treatment of major depressive disorder.^23^ Fluvoxamine, a selective serotonin reuptake inhibitor (SSRI) and S1R agonist, has shown promise in the prevention of COVID-19 progression as an early treatment option in phase 2 clinical trial.^24^ The S1R radioligand [^18^F]FTC-146 has demonstrated potential for validating the correlation between S1R distribution and pathologies of different etiology.^25−29^ S2R is a poorly understood protein identified as an endoplasmic reticulum-resident transmembrane protein (TMEM97) playing a role in the cholesterol homeostasis and the sterol transporter Niemann–Pick disease type C1.^30^ High levels of S2R have been found in several cancer cells and proliferating tumors, including lung, breast, and colorectal cancer.^31^ In this context, S2R has been proposed as drug target for the diagnosis and treatment of cancer and radiotracers with affinity for S2R have been developed as tumor imaging agents.^32−34^ Compound [^18^F]-ISO-1 is a positron emission tomography (PET) ligand evaluated in clinical trials for the imaging of S2R binding in primary breast cancer.^26^ Specifically, the activation of S2R/TMEM97 promotes cancer cell apoptosis resulting in anticancer activity,^35^ while the inhibition or antagonism of the S2R/TMEM97 has a role in neuroprotection, neurodegeneration, improved cognition, and anti-dementia. Elayta (CT1812), a small-molecule S2R antagonist which binds to the receptors at the progesterone receptor membrane component 1 subunit, is currently under phase II clinical trial in patients with mild to moderate Alzheimer’s disease.^36,37^ Ligands for the S2R/PGRMC1 receptor are reported to be negative allosteric regulators that reduce the affinity of oligomeric Aβ for its receptor, and thus interfere with Aβ-induced synaptic toxicity.^38^ Also, roluperidone (MIN-101) is a compound with S2R, 5-HT_2A_ and α_1A_-adrenergic receptor antagonist profile in phase III clinical trials for the treatment of negative symptoms of schizophrenia.^39,40^

**Figure 2:**
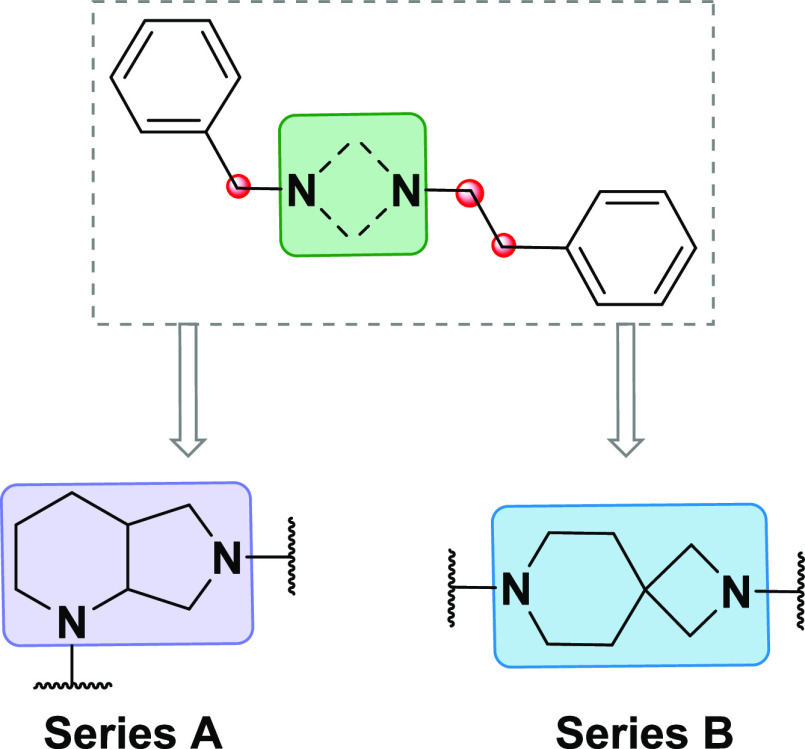
General structure of designed SR ligands and sites of structure–activity relationship (SAR) exploration.

Recently, our group has synthesized and biologically evaluated a library of SR ligands based on 2,7-diazaspiro[4.4]nonane scaffold, finding out a compound with extreme potency in a model of capsaicin-induced allodynia without side effects on motor function.^41^ Following up our studies in this field, herein we report the development of a new class of SR ligands designed through the modification of the amino moiety scaffold used for our previously synthesized ligands. Thus, diazabicyclo[4.3.0]nonane (A) and 2,7-diazaspiro[3.5]nonane (B) derivatives have been designed (Figure 2), where the central core has been flanked with hydrophobic groups at a certain distance to the central basic amine being this a common structural requirement of potent SR ligands as identified by previous works.^42−47^ A total of 15 compounds with varying distal hydrophobic region and linker portion were evaluated. Herein, we describe the SAfiR studies and subtype preference derived from radioligand binding, the binding mode and the interactions established between the ligands and the S1R and S2R. Moreover, we report the *in vivo* pharmacological effects in a model of mechanical hypersensitivity in order to further discover the impact of chemical modifications on the functional character.

## Results and Discussion

### Chemistry

**Scheme 1:**
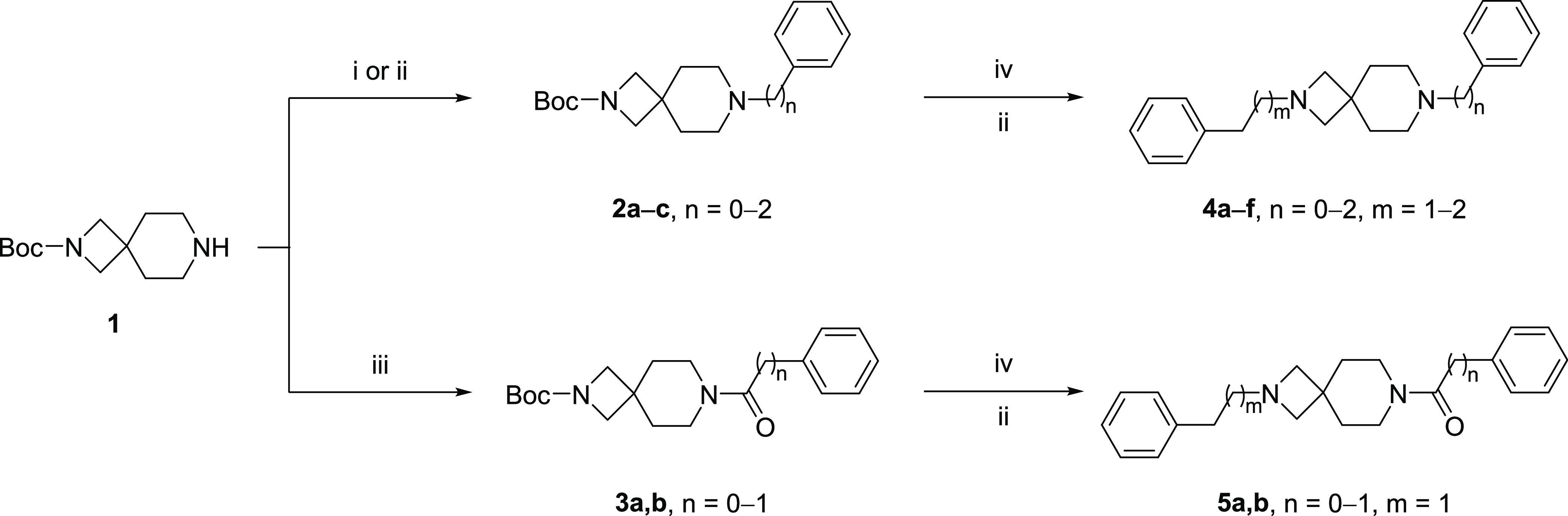
Synthetic Strategy for the Preparation of Target Compounds **4a**–**f** and **5a**,**b** Reagents and conditions: (i) iodobenzene, Pd_2_(dba)_3_, SPhos, *t*-BuOK, toluene, 100 °C, on; (ii) alkyl bromide, K_2_CO_3_, ACN, 50 °C, on; (iii) acyl chloride, triethylamine (TEA), CH_2_Cl_2_, rt, 2 h; (iv) TFA, CH_2_Cl_2_, rt, 4 h; (iv) Boc_2_O, CH_2_Cl_2_, rt, on.

**Scheme 2:**
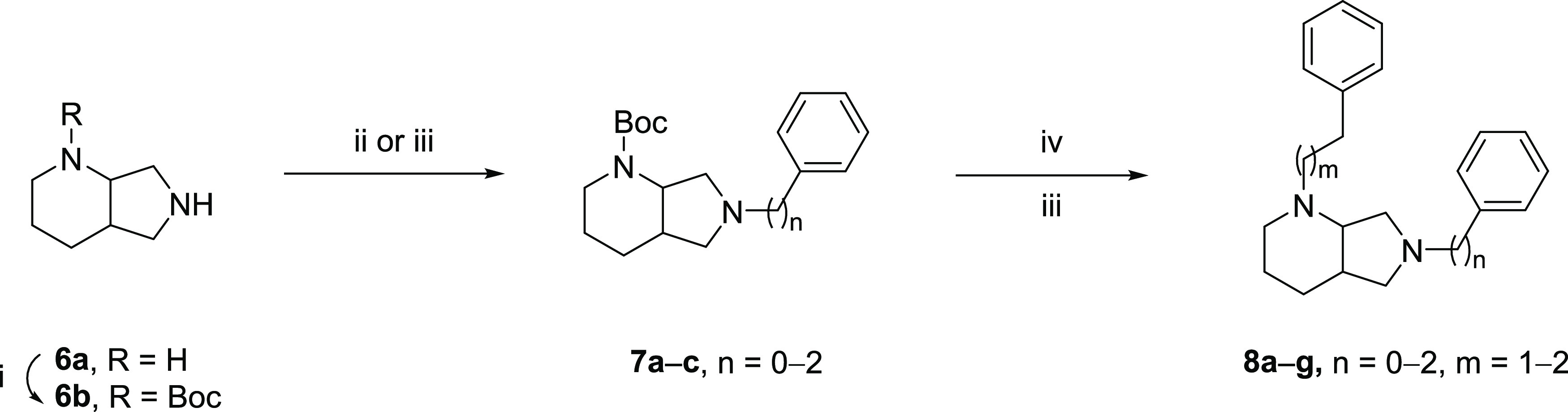
Synthetic Strategy for the Preparation of Target Compounds **8a**–**g** Reagents and conditions: (i) Boc_2_O, CH_2_Cl_2_, rt, on; (ii) iodobenzene, Pd_2_(dba)_3_, SPhos, *t*-BuOK, toluene, 100 °C, on; (iii) alkyl bromide, K_2_CO_3_, ACN, 50 °C, on; (iv) TFA, CH_2_Cl_2_, rt, 4 h.

The synthesis of the new compounds is depicted in Schemes 1 and 2. Starting from the commercially available *tert*-butyl 2,7-diazaspiro[3.5]nonane-2-carboxylate (**1**), intermediate **2a** has been obtained via Buchwald–Hartwig amination with iodobenzene, followed by *N*-Boc deprotection and subsequent alkylation with either benzylbromide or (2-bromoethyl)benzene, which provided rapid access to the desired compounds **4a** and **4d**. Alkylation of **1** with either benzylbromide or (2-bromoethyl)benzene gave intermediates **2b**,**c**, whereas nucleophilic acyl substitution with benzoyl chloride or 2-phenylacetyl chloride gave the corresponding final compounds **3a**,**b**. All of the intermediates were deprotected with trifluoroacetic acid (TFA), followed by alkylation with (2-bromoethyl)benzene or 1-bromo-3-phenylpropane to give the corresponding final compounds **4b**,**c**, **4e**,**f**, and **5a**,**b**.

According to the steps illustrated in Scheme 2, the commercially available (*S*,*S*)-2,8-diazabicyclo[4.3.0]nonane (**6a**) was previously *N*-protected with Boc anhydride to give the amine **6b**. Intermediate **7a** was obtained via Buchwald–Hartwig amination with iodobenzene, whereas derivatives **7b**,**c** were obtained by alkylation with (2-bromoethyl)benzene or 1-bromo-3-phenylpropane. All of the intermediates were deprotected with TFA, followed by a final alkylation providing the desired final compounds **8a**–**g**.

### Binding Properties and SAfiR Analysis

All of the synthesized compounds were evaluated for affinity at both S1R and S2R through radioligand binding assays. The 2,7-diazaspiro[3.5]nonane derivatives have revealed a range of different *K*_i_ values for S1R (Table 1). In particular, compounds containing two basic nitrogen showed low nanomolar *K*_i_ for S1R with values of 2.7 and 3.5 nM for **4b**,**c**, respectively, and 6- to 10-fold preference over S2R. Compound **4a**—bearing the phenyl ring directly joined to the nitrogen atom and a *N*-phenethyl group on the other side—slightly lost affinity over both receptors compared to **4b**,**c** values, but still in the double-digit nanomolar range. Similar profile has been shown for compound **4d** having analogue structure but a three-carbon chain on the basic nitrogen. The reinstatement of the basic nitrogen as in **4e** brought back S1R affinity to the low nanomolar value of 7.2 nM and a slight preference over S2R. Further elongation of carbon chain on both nitrogen atoms as in compound **4f** determined a decrease of affinity on both subtypes. Variations of **4b** as in compound **5b** determined a weak reduction in both SRs affinity, with a sequential maintenance of S1R affinity preference. Modification of **4c** with an amide function resulted in **5a** with ∼10-fold reduced affinity on both subtypes.

**Table 1:**
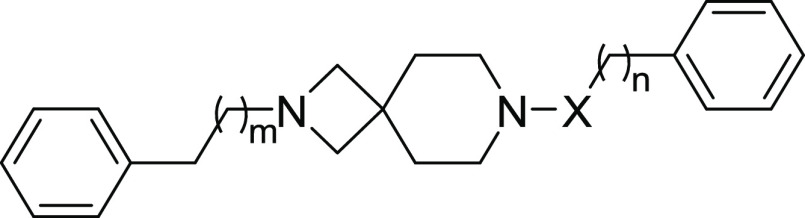
Affinities for 2,7-Diazaspiro[3.5]nonane Derivatives

compound	n	m	X	*K*_i_ (nM) ± SD^a^		
				S1R	S2R	*K*_i_S2R/*K*_i_S1R
**4a**	0	1	-	28 ± 2.0	77 ± 12	2.8
**4b**	1	1	-	2.7 ± 0.3	27 ± 5.3	10
**4c**	2	1	-	3.5 ± 0.3	22 ± 2.5	6.3
**4d**	0	2	-	34 ± 7.7	51 ± 5.0	1.5
**4e**	1	2	-	7.2 ± 0.5	31 ± 5.4	4.3
**4f**	2	2	-	126 ± 22	149 ± 27	1.2
**5a**	0	1	CO	37 ± 9.6	255 ± 62	6.8
**5b**	1	1	CO	13 ± 2.5	102 ± 26	7.8
**(+)-Pentazocine**				4.3 ± 0.5	1465 ± 224	
**DTG**				124 ± 19	18 ± 1	

a Each value is the mean ± standard deviation (SD) of at least two experiments performed in duplicate.

In the series of diazabicyclo[4.3.0]nonane, all of the compounds showed a general loss of affinity on both receptors (Table 2), with the only exceptions of **8c** and **8f**. Compound **8c**—bearing a benzyl group on the five-membered ring and a phenethyl on the six-membered—has a *K*_i_ of 19 for S1R and more than 7-fold preference over S2R. Also **8f**—with a further methylene group on both sides—showed lower double-digit *K*_i_S1R value (10 nM), and improved preference over S2R. Conversely, keeping the chain length on the six-membered ring and reducing the one on the five-membered (**8d**) determined an inversion of SR profile with negligible *K*_i_S1R values. The presence of a plain phenyl ring directly joined to the five-membered ring (**8a**, **8b**, **8e**)—therefore having only one nitrogen able to create ionic interactions with the targets—determined an S2R preferential affinity. The symmetric derivative **8g** shows an affinity reduction for both SR, although a slightly S1R preferential affinity seems to be restored.

**Table 2:**
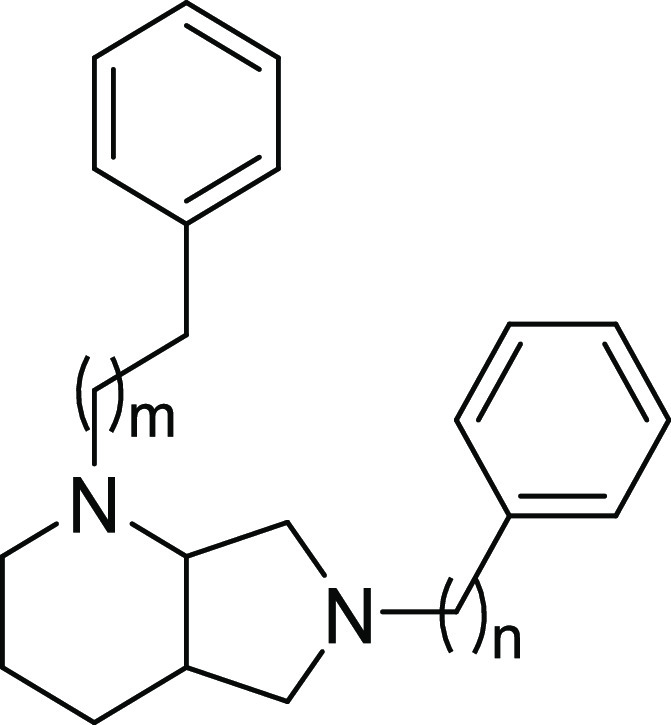
Affinities for Diazabicyclo[4.3.0]nonane Derivatives

			*K*_i_ (nM) ± SD^a^		
compound	n	m	S1R	S2R	*K*_*i*_S2R/*K*_i_S1R
**8a**	0	0	254 ± 32	148 ± 29	0.6
**8b**	0	1	505 ± 88	279 ± 83	0.6
**8c**	1	1	19 ± 2.6	126 ± 19	6.6
**8d**	2	1	1726 ± 403	114 ± 15	0.07
**8e**	0	2	108 ± 31	91 ± 5.8	0.8
**8f**	1	2	10 ± 1.6	165 ± 36	16.5
**8g**	2	2	710 ± 150	1026 ± 200	1.4

a Each value is the mean ± SD of at least two experiments performed in duplicate.

Overall, the 2,7-diazaspiro[3.5]nonane moiety has provided compounds with optimal features for SR recognition allowing us to achieve high-affinity ligands. Two compounds, **4b** and **5b**, have emerged as S1R high-affinity ligands with preference over S2R in the 10-fold range. On the other hand, among the diazabicyclo[4.3.0]nonane derivative **8f** has emerged as high-affinity S1R ligand.

### Molecular Modeling

Molecular docking simulations highlighted that all of the compounds are able to recognize and bind both S1R and S2R binding pockets. In fact, they are well accommodate in the S1R and S2R binding sites interacting with the pivotal amino acid residues of the binding pockets, and they are associated to a good theoretical binding affinity (Table 3).

**Table 3:** Docking Score Values of the Studied Compounds in the Presence of S1R and S2R

	docking score values^a^	
compound	S1R	S2R
**4a**	–8.21	–7.85
**4b**	–8.84	–8.02
**4c**	–8.64	–8.21
**4d**	–9.22	–8.25
**4e**	–9.33	–8.01
**4f**	–9.45	–8.56
**5a**	–9.45	–8.31
**5b**	–9.72	–8.32
**8a**	–7.77	–7.28
**8b**	–9.22	–7.33
**8c**	–8.32	–8.31
**8d**	–9.76	–7.49
**8e**	–9.47	–8.04
**8f**	–9.81	–7.63
**8g**	–10.28	–6.92

a Expressed in kcal/mol.

Regarding SR1, all of the compounds are able to establish a salt bridge and/or a hydrogen-bond interaction with the Glu172, a highly conserved residue located near the center of the cavity. In addition, π–π stacking interactions are established between the benzyl rings of some ligands and the Tyr103, Phe133, and His154.

**Figure 3:**
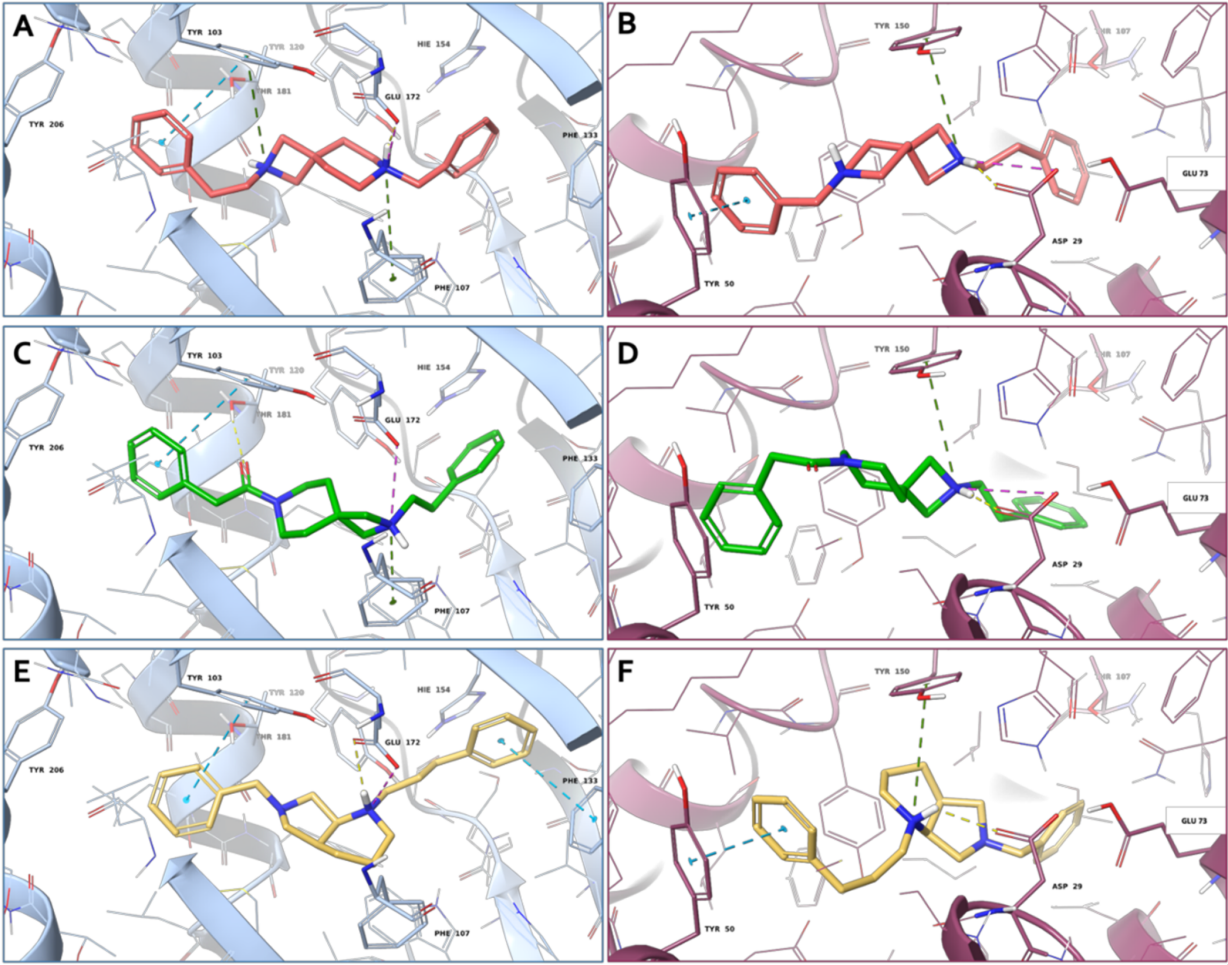
3D representation of (A, B) compound **4b** (salmon carbon sticks), (C, D) compound **5b** (green carbon sticks) and (E, F) compound **8f** (yellow carbon sticks) in complex with S1R (A, C, E) and S2R (B, D, F), respectively. The S1R and S2R are represented as blue and violet cartoons, respectively. The receptor residues involved in crucial contacts with the compounds are reported as blue and violet carbon sticks. Hydrogen bonds, salt bridges, π–π stacking, and π-cation interactions are represented by yellow, magenta, azure, and green dashed lines, respectively.

For some compounds, the protonated nitrogen on the diazabicyclo core makes a π-cation with the Phe107, while the other nitrogen, if charged, is involved in another π-cation interaction with Tyr103. Furthermore, the carbonyl group of compounds **5a** and **5b** is engaged in a hydrogen bond with Thr181. Moreover, the compounds establish several hydrophobic contacts with the residues that coat the inner walls of the binding cavity. The three-dimensional (3D) representations of the most promising compounds are reported in Figure 3A,C,E.

Considering the SR2, all of the ligands lie in the receptor binding site establishing different kinds of interactions with the key residues of the pocket. In detail, all of the compounds establish, through their protonated nitrogen atom, a hydrogen bond and a salt bridge interaction with the conserved acid residue Asp29, and for some ligands, the same nitrogen atom is engaged in a π–cation with the Tyr150. In some cases, the benzyl ring of the compounds is engaged in a π–π stacking with Tyr50, His21, and Tyr150. Moreover, all of the ligands are well stabilized in the SR2 by means of several hydrophobic residues. The 3D representations of the most promising compounds are reported in Figure 3B,D,F.

### Effects of **4b**, **5b**, and **8f** on Capsaicin-Induced Mechanical Hypersensitivity

We tested the effects of compounds **4b**, **5b**, and **8f** on sensory hypersensitivity in mice, using BD-1063 and PRE-084 as the prototypes of S1R antagonist and agonist, respectively. These three compounds have been selected for their higher S1R affinity and preference over S2R subtype. Indeed, compound **4b** is the compound with the lowest S1R affinity of the plain 2,7-diazaspiro[3.5]nonane series while compound **5b**—bearing an amide function—is the highest-affinity ligand of the 2,7-diazaspiro[3.5]nonane series where just one nitrogen is able to establish a salt bridge and/or a hydrogen-bond interaction with the Glu172. At the same time, compound **8b** is the highest-affinity compound in the diazabicyclo[4.3.0]nonane series. Considering that the 2,7-diazaspiro[3.5]nonane and the diazabicyclo[4.3.0]nonane are new scaffolds for SR ligands, we were wondering about the functional profile and thus the potential effects that these molecules might exert in an *in vivo* pain model.

We used capsaicin-induced secondary hypersensitivity (allodynia) as a pain model. It has been described that the increase in mechanical sensitivity in the area surrounding capsaicin injection (area of secondary allodynia) is due to central sensitization, which is a relevant process for chronic pain development and maintenance.^48^ This pain model has been previously used to determine the S1R agonistic/antagonistic properties of new compounds (including the clinical candidate S1RA), as S1R antagonists decrease mechanical hypersensitivity while S1R agonists reverse the effects of the former.^41,49−51^

**Figure 4:**
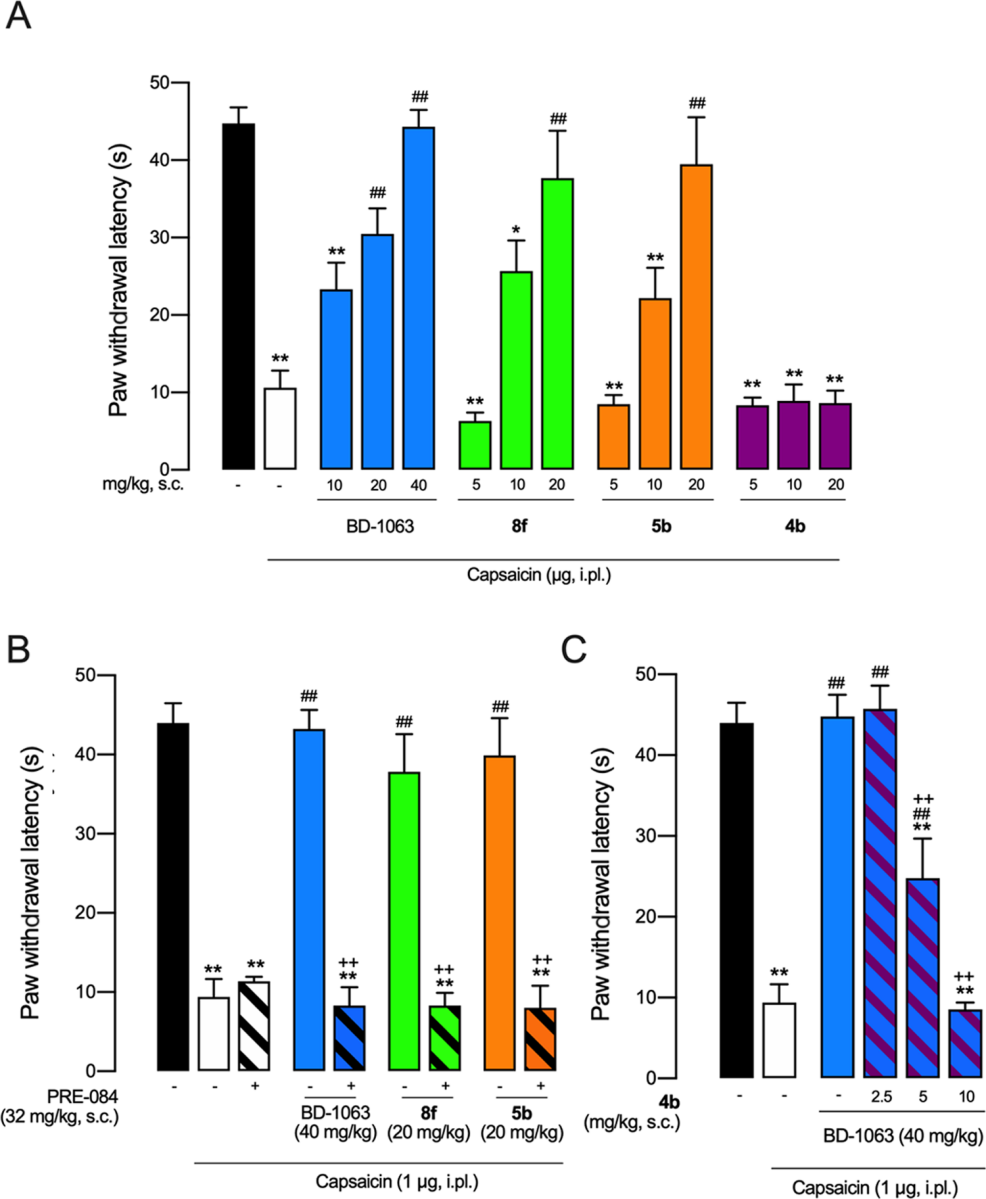
Effects of the systemic administration of standard S1R drugs (BD-1063 and PRE-084) and several experimental compounds in capsaicin-induced mechanical hypersensitivity in mice. (A) Effects of the subcutaneous (s.c.) administration of BD-1063, **8f** and **5b** on mechanical allodynia induced by the intraplantar (i.pl.) administration of capsaicin (1 μg), and absence of antiallodynic effects induced by **4b**. (B) Effects of BD-1063, **8f** and **5b** administered alone and associated with the S1R agonist PRE-084. (C) Effect of BD-1063 alone and associated with **4b**. Values are the mean ± standard error of the mean (SEM) obtained from six to nine animals per group: * *p* < 0.05, **p < 0.01 *vs* nonsensitized animals treated with the solvent of the drugs (black bar); ^#^*p* < 0.05, ^##^*p* < 0.01 *vs* capsaicin-injected mice treated with the solvent of the drugs (white bar); ^+^*p* < 0.05,^*++*^*p* < 0.01 selected doses of antiallodynic compounds associated with PRE-084 (B) or **4b** (C), or their solvent (one-way analysis of variance (ANOVA) followed by Bonferroni test).

Intraplantar (i.pl.) administration of 1 μg of capsaicin resulted in a marked reduction in the response latency to mechanical stimulation (black *vs* white bars in Figure 4A), denoting the presence of mechanical hypersensitivity, as expected. Subcutaneous (s.c.) administration of BD-1063 resulted in a full and dose-dependent reduction of mechanical hypersensitivity, and administration of either **8f** or **5b** mimicked the effect of the known S1R antagonist (Figure 4A). However, both **8f** and **5b** exhibited higher potency than BD-1063. Although BD-1063 needed 40 mg/kg to achieve full reversal of mechanical hypersensitivity, **8f** and **5b** needed half of this dose to reach a similar effect (Figure 4A). On the other hand, the administration of **4b** (5–20 mg/kg) was completely devoid of antiallodynic effect (Figure 4A).

We then compared the effect of s.c. doses of BD-1063, **8f** and **5b** that were able to fully reverse mechanical allodynia (40 mg/kg of BD-1063 and 20 mg/kg of the two newly synthesized compounds) alone or associated with the S1R agonist PRE-084 (32 mg/kg, s.c.). PRE-084 did not modify the response latency of capsaicin-injected mice treated with the solvent of the drugs but was able to abolish the antiallodynic effect of not only the standard BD-1063 but also that induced by **8f** and **5b** (Figure 4B). The similarities in the effects induced by the known S1R antagonist BD-1063 with **8f** and **5b** (i.e., a prominent antiallodynic effect reversed by the S1R agonist PRE-084), clearly indicate that both **8f** and **5b** induce an S1R antagonistic *in vivo* effect.

Since **4b** showed high affinity for S1R but—as described above—no antiallodynic effect, we hypothesized that this compound might behave as an S1R agonist, in contrast to the other tested compounds. Therefore, we associated a s.c. dose of BD-1063 which induced a marked antiallodynic effect (40 mg/kg) with several doses of **4b** (2.5–10 mg/kg, s.c.), and found that the latter was able to fully reverse the antiallodynic effect of BD-1063 (Figure 4C). The similarities between the effects induced by the known S1R agonist PRE-084 with **4b** (i.e., no antiallodynic effect when administered alone but able to fully reverse the effect induced by S1R antagonists), clearly indicate that **4b** induces an S1R agonistic *in vivo* effect.

### *In Vitro* Functional Profile

In order to further asses the functional profile of compounds **4b**, **5b**, and **8f**, the well-established phenytoin-based functional assay was carried out.^52^ Previous studies have shown that phenytoin, a low-potent allosteric modulator for the S1R, differentially modulates the affinity of S1R ligands depending on their agonist *versus* antagonist functionality. Phenytoin potentiates the receptor binding affinity of S1R agonists (ratio *K*_i_ control/with phenytoin >1); however, it produces no effects or slightly reduced receptor binding affinity for S1R antagonists (ratio *K*_i_ control/with phenytoin ≤1).

In a similar manner to the known S1R antagonist BD-1063, both **5b** and **8f** exhibited a very small shift to lower receptor binding affinity with ratios of 0.9 and 0.8, respectively. These observations indicated that both compounds **5b** and **8f** act as antagonists for the S1R. On the contrary, compound **4b** and SKF-10,047 showed ratios of 2.6 and 5.8, confirming the agonist profile for the S1R (Table 4).

**Table 4:** Ratio of *K*_i_ Values with or without Phenytoin in the S1R Radioligand Binding Assay

compound	*K*_i_ (nM) control	*K*_i_ (nM) + phenythoin	ratio *K*_i_ (nM) control/+ phenythoin
**4b**	3.2	1.2	2.6
**5b**	12	14	0.9
**8f**	9.6	12	0.8
**SKF-10,047**	99	17	5.8
**BD-1063**	6.7	10	0.7

## Conclusions

Here we report the design and synthesis of a series of diazabicyclo[4.3.0]nonane and 2,7-diazaspiro[3.5]nonane derivatives as SR ligands. Overall, the 2,7-diazaspiro[3.5]nonane series has given compounds with higher binding affinity with respect to the diazabicyclo[4.3.0]nonane. Three compounds, **4b** (*K*_i_S1R = 2.7 nM, *K*_i_S2R = 27 nM), **5b** (*K*_i_S1R = 13 nM, *K*_i_S2R = 102 nM), and **8f** (*K*_i_S1R = 10 nM, *K*_i_S1R = 165 nM), have been selected to be screened for antiallodinic effects *in vivo* and to determine their functional profiles through *in vivo* and *in vitro* models. Compounds **5b**, and **8f** reached the maximum antiallodynic effect at 20 mg/Kg doses, achieving the full reversal of mechanical hypersensitivity at half of the dose of the prototypical S1R antagonist BD-1063 (40 mg/kg). The selective S1R agonist PRE-084 completely reversed their antiallodynic action, indicating that their effects are entirely dependent on the S1R antagonism profile. Conversely, compound **4b** sharing the 2,7-diazaspiro[3.5]nonane core as **5b**, was completely devoid of antiallodynic effect. Interestingly, compound **4b** was able to fully reverse the antiallodynic effect of BD-1063, clearly indicating that **4b** induces an S1R agonistic *in vivo* effect. The functional profiles were confirmed by the phenytoin S1R binding assay, indicating that compounds **5b** and **8f** are S1R antagonists while compound **4b** is an S1R agonist since its *K*_i_ in the presence of the allosteric modulator phenytoin is 2.6-fold higher than the *K*_i_ determined without modulator. Furthermore, the binding mode analysis of compounds **4b** and **5b** in S1R binding pocket revealed that the two compounds interact with the receptor in a different way. Indeed, in compound **4b**, the piperidine nitrogen establishes a salt bridge interaction and a hydrogen bond with the highly conserved residue Glu172; meanwhile, in compound **5b**, the nitrogen of the azetidine engaged in a salt bridge interaction with Glu172.

In conclusion, the present study provided new insights into the use of 2,7-diazaspiro[3.5]nonane and diazabicyclo[4.3.0]nonane scaffolds in the development of novel SR ligands. The differences in functional profile for compounds **4b** and **5b**, sharing the 2,7-diazaspiro[3.5]nonane scaffold, give rise to the opportunity for future investigation to determine the structural features which relate with the intrinsic activity.

## Methods

### General Details

Reagent grade chemicals were purchased from Sigma-Aldrich (St. Louis, Missouri) or Merck (Darmstadt, Germany) and were used without further purification. All reactions involving air-sensitive reagents were carried out under nitrogen in dried glassware using the syringe-septum cap technique. Flash chromatography purification was performed on a Merck silica gel 60 (40–63 μm; 230–400 mesh) stationary phase. Nuclear magnetic resonance spectra (^1^H NMR recorded at 200 and 500 MHz) were obtained on VARIAN INOVA spectrometers using CDCl_3_, DMSO-*d*_6_, or CD_3_OD. TMS was used as an internal standard. Chemical shifts (δ) are given in parts per million (ppm) and coupling constants (*J*) in hertz (Hz). The following abbreviations are used to designate the multiplicities: s = singlet, d = doublet, t = triplet, m = multiplet, br = broad. The purity of all tested compounds, whether synthesized or purchased, reached at least 95% as determined by microanalysis (C, H, N) that was performed on a Carlo Erba instrument model E1110; all of the results agreed within ±0.4% of the theoretical values. Reactions were monitored by thin-layer chromatography (TLC) performed on 250 μm silica gel Merck 60 F_254_-coated aluminum plates; the spots were visualized by UV light or iodine chamber. UV–vis spectra absorption spectra were recorded with a Jasco V-560 spectrophotometer. Compound nomenclatures were generated with ChemBioDraw Ultra version 16.0.0.82.

#### General Procedure for Buchwald–Hartwig Amination (Procedure A)

A mixture of Pd_2_(dba)_3_ (3 mol %, 11 mg) and 2-dicyclohexylphosphino-2′,6′-dimethoxybiphenyl (8 mol %, 13 mg) in dry toluene (3 mL) was degassed under N_2_ for 30 min into an oven-dried sealed vial. Then, para-substituted iodobenzene (0.39 mmol), *tert*-butyl 2,7-diazaspiro[3.5]nonane-2-carboxylate or *tert*-butyl octahydro-1*H*-pyrrolo[3,4-*b*]pyridine-1-carboxylate (**6b**, 0.39 mmol), and *t*-BuONa (0.55 mmol, 62 mg) were sequentially added, and the reaction was stirred. The reaction mixture was slowly brought to rt, quenched with H_2_O (5 mL), and extracted with EtOAc (2 × 10 mL). The organic layer was dried over Na_2_SO_4_ and concentrated under vacuum. The crude product was purified by flash chromatography on silica gel to afford the desired product.

#### General Procedure for Amine Preparation (Procedure B)

To a solution of *tert*-butyl 2,7-diazaspiro[3.5]nonane-2-carboxylate or *tert*-butyl octahydro-1*H*-pyrrolo[3,4-*b*]pyridine-1-carboxylate (**6b**, 0.44 mmol) in ACN (5 mL), K_2_CO_3_ (1.32 mmol, 182 mg) and halo-derivative (0.73 mmol) were sequentially added. The reaction was stirred for 3 h at rt, then quenched with H_2_O (5 mL), and extracted with EtOAc (2 × 10 mL). The organic layer was washed with brine (1 × 5 mL), dried over Na_2_SO_4_, filtered, and concentrated under vacuum. The residue was purified via silica gel chromatography to obtain the desired product.

#### General Procedure for Amine Preparation (Procedure C)

The Boc-protected amine (0.2 mmol) was stirred with 30% TFA in CH_2_Cl_2_ (10 mL) at rt for 4 h, followed by the removal of the solvents *in vacuo*. The residue was then dissolved in ACN (5 mL), and K_2_CO_3_ (0.3 mmol, 41 mg) and alkyl bromide (0.2 mmol) were sequentially added. The reaction was stirred under reflux, quenched with H_2_O (5 mL), and extracted with EtOAc. The collected organic phases were washed with brine (1 × 5 mL), dried over Na_2_SO_4_, and evaporated to dryness.

#### General Procedure for Amine Preparation (Procedure D)

To a solution of *tert*-butyl 2,7-diazaspiro[3.5]nonane-2-carboxylate (0.44 mmol, 100 mg) in anhydrous CH_2_Cl_2_ (5 mL), TEA (0.66 mmol, 92.41 μL) and acyl chloride (0.88 mmol) were added dropwise at 0 °C. The reaction was stirred for 1 h at rt, then quenched with cold water (5 mL), diluted with CH_2_Cl_2_ (10 mL), and washed with 5% NH_4_Cl (1 × 5 mL) and a saturated solution of NaHCO_3_ (1 × 5 mL). The combined organic extracts were dried over anhydrous Na_2_SO_4_ and concentrated under vacuum. The residue was purified by flash chromatography.

#### General Procedure for Amine Preparation (Procedure E)

A mixture of Boc-protected amine (0.30 mmol) with 30% TFA in CH_2_Cl_2_ (10 mL) was stirred at rt for 4 h, followed by the removal of the solvents *in vacuo*. The residue was dissolved in fresh anhydrous CH_2_Cl_2_ (5 mL), and TEA (0.53 mmol, 75 μL) and acyl chloride (0.36 mmol) were added dropwise at 0 °C. The reaction was stirred at rt for 2 h and then quenched with H_2_O (5 mL), diluted with CH_2_Cl_2_ (10 mL), and washed with 5% NH_4_Cl (1 × 5 mL) and then with a saturated solution of NaHCO_3_ (1 × 5 mL). The combined organic extracts were dried over anhydrous Na_2_SO_4_ and concentrated under vacuum. The residue was purified by flash chromatography to obtain the desired product.

#### General Procedure for Oxalate Salt Formation (Procedure F)

For all of the final compounds, the pure product was dissolved in diethyl ether and a solution of oxalic acid in diethyl ether was added dropwise to obtain the desired product as an oxalic acid salt.

##### *tert*-Butyl 7-Phenyl-2,7-diazaspiro[3.5]nonane-2-carboxylate (**2a**)

The compound was prepared using *tert*-butyl 2,7-diazaspiro[3.5]nonane-2-carboxylate (0.39 mmol, 88 mg) and iodobenzene (0.39 mmol, 43.6 μL) following Procedure A. The crude product was purified by column chromatography on silica gel using hexane/EtOAc (90:10) as the eluent to afford the desired product. Yield: 89%, clear solid. ^1^H NMR (200 MHz, CDCl_3_) δ 7.16–7.33 (m, 2H), 6.76–6.98 (m, 3H), 3.66 (s, 4H), 2.94–3.22 (m, 4H), 1.79–1.91 (m, 4H), 1.45 (s, 9H).

##### *tert*-Butyl 7-Benzyl-2,7-diazaspiro[3.5]nonane-2-carboxylate (AD179, **2b**)

The compound was prepared using *tert*-butyl 2,7-diazaspiro[3.5]nonane-2-carboxylate (0.44 mmol, 100 mg) and benzylbromide (0.73 mmol, 86.8 μL) following Procedure B. The residue was purified via silica gel chromatography with 100% EtOAc. Yield: 70%, clear oil. ^1^H NMR (200 MHz, CDCl_3_) δ 7.08–7.51 (m, 5H), 3.41–3.72 (m, 6H), 2.40 (br. s., 4H), 1.68–1.98 (m, 4H), 1.36 (s, 9H).

##### tert-Butyl 7-Phenethyl-2,7-diazaspiro[3.5]nonane-2-carboxylate (AD178, **2c**)

The compound was prepared using *tert*-butyl 2,7-diazaspiro[3.5]nonane-2-carboxylate (0.44 mmol, 100 mg) and (2-bromoethyl)benzene (0.73 mmol, 134 μL) following Procedure B. The residue was purified with 5% MeOH in EtOAc. Yield: 41%, yellow oil. ^1^H NMR (200 MHz, CDCl_3_) δ 7.13–7.40 (m, 5H), 3.63 (s, 4H), 2.73–2.91 (m, 2H), 2.33–2.65 (m, 6H), 1.80 (t, *J* = 5.5 Hz, 4H), 1.46 (s, 9H).

##### 2-Phenethyl-7-phenyl-2,7-diazaspiro[3.5]nonane (**AD172, 4a**)

The compound was prepared using **2a** (0.20 mmol, 60 mg) and (2-bromoethyl)benzene (0.20 mmol, 27.3 μL) following Procedures C and F. The residue was purified by flash chromatography on silica gel eluting with 3% MeOH in CH_2_Cl_2_. Yield: 30%, yellow solid. ^1^H NMR (200 MHz, CDCl_3_—free base) δ 7.20–7.41 (m, 7H), 6.79–7.00 (m, 3H), 3.30 (s, 4H), 3.05–3.18 (m, 4H), 2.72–3.00 (m, 4H), 1.89–2.00 (m, 4H). ^13^C NMR (200 MHz, CDCl_3_—free base) δ 147.7, 140.1, 129.2, 128.7, 128.3, 126.1, 115.4, 111.4, 64.8, 59.6, 58.4, 54.2, 48.0, 47.1, 37.9, 36.0, 35.4. Elem. Anal. for C_21_H_26_N_2_·H_2_C_2_O_4_, calcd: C, 69.68; H, 7.12; N, 7.07; found: C, 69.92; H, 7.17; N, 7.06.

##### 7-Benzyl-2-phenethyl-2,7-diazaspiro[3.5]nonane (**AD186**, **4b**)

The compound was prepared using **2b** (0.27 mmol, 80 mg) and (2-bromoethyl)benzene (0.32 mmol, 44 μL) following Procedures C and F. The residue was purified by flash chromatography on silica gel eluting with 4% MeOH in CH_2_Cl_2_. Yield: 30%, white solid. ^1^H NMR (200 MHz, CDCl_3_—free base) δ 7.09–7.37 (m, 10H), 3.43 (s, 2H), 3.13 (s, 4H), 2.61–2.89 (m, 4H), 2.31 (br. s., 4H), 1.77 (t, *J* = 5.4 Hz, 4H). ^13^C NMR (200 MHz, CDCl_3_—free base) δ 139.1, 138.1, 129.1, 128.5, 128.1, 127.0, 126.3, 64.3, 63.2, 61.0, 50.4, 35.9, 34.2, 33.8. Elem. Anal. for C_22_H_28_N_2_·H_2_C_2_O_4_, calcd: C, 70.22; H, 7.37; N, 6.82; found: C, 70.53; H, 7.42; N, 6.76.

##### 2,7-Diphenethyl-2,7-diazaspiro[3.5]nonane (**AD187**, **4c**)

The compound was prepared using **2c** (0.20 mmol, 66 mg) and (2-bromoethyl)benzene (0.20 mmol, 36.8 μL) following Procedures C and F. The residue was purified by flash chromatography on silica gel eluting with 4% MeOH in CH_2_Cl_2_. Yield: 25%, white solid. ^1^H NMR (200 MHz, CDCl_3_—free base) δ 7.09–7.36 (m, 10H), 2.99–3.16 (m, 6H), 2.62–2.88 (m, 6H), 2.31–2.61 (m, 6H), 1.81 (t, *J* = 4.9 Hz, 2H). ^13^C NMR (200 MHz, CDCl_3_—free base) δ 140.1, 139.2, 128.8, 128.4, 126.3, 126.1, 64.3, 61.2, 60.7, 50.6, 35.9, 34.2, 33.9, 33.6. Elem. Anal. for C_23_H_30_N_2_·H_2_C_2_O_4_, calcd: C, 70.73; H, 7.60; N, 6.60; found: C, 70.97; H, 7.65; N, 6.59.

##### 7-Phenyl-2-(3-phenylpropyl)-2,7-diazaspiro[3.5]nonane (**AD173, 4d**)

The compound was prepared using **2a** (0.20 mmol, 60 mg) and (3-bromopropyl)benzene (0.20 mmol, 30.4 μL) following Procedures C and F. The residue was purified by flash chromatography on silica gel eluting with 5% MeOH in CH_2_Cl_2_. Yield: 30%, brown solid. ^1^H NMR (200 MHz, CDCl_3_—free base) δ 7.10–7.45 (m, 7H), 6.78–6.99 (m, 3H), 3.70 (s, 4H), 3.04–3.18 (m, 4H), 2.92–3.04 (m, 2H), 2.72 (t, *J* = 7.4 Hz, 2H), 1.94–2.15 (m, 6H). ^13^C NMR (200 MHz, CDCl_3_—free base) δ 137.2, 136.3, 129.2, 128.8, 128.6, 128.5, 127.8, 126.9, 64.9, 64.5, 59.5, 57.7, 53.7, 52.8, 47.7, 37.1, 36.8, 32.9. Elem. Anal. for C_22_H_28_N_2_·H_2_C_2_O_4_, calcd: C, 70.22; H, 7.37; N, 6.82; found: C, 70.56; H, 7.39; N, 6.85.

##### 7-Benzyl-2-(3-phenylpropyl)-2,7-diazaspiro[3.5]nonane (**AD195**, **4e**)

The compound was prepared using **2b** (0.20 mmol, 63 mg) and (3-bromopropyl)benzene (0.20 mmol, 30.4 μL) following Procedures C and F. The residue was purified by flash chromatography on silica gel eluting with 4% MeOH in CH_2_Cl_2_. Yield: 35%, yellow solid. ^1^H NMR (200 MHz, CDCl_3_—free base) δ 7.10–7.46 (m, 10H), 3.80 (s, 2H), 3.69 (s, 2H), 3.49 (s, 2H), 2.18–2.56 (m, 4H), 1.80 (t, *J* = 5.4, 4H), 1.65 (br. s., 6H). ^13^C NMR (200 MHz, CDCl_3_—free base) δ 143.2, 138.0, 129.0, 128.5, 128.4, 128.2, 127.1, 63.0, 58.0, 56.9, 50.3, 47.7, 35.4, 29.7. Elem. Anal. for C_23_H_30_N_2_·H_2_C_2_O_4_, calcd: C, 70.73; H, 7.60; N, 6.60; found: C, 70.87; H, 7.62; N, 6.58.

##### 7-Phenethyl-2-(3-phenylpropyl)-2,7-diazaspiro[3.5]nonane (**AD197**, **4f**)

The compound was prepared using **2c** (0.20 mmol, 66 mg) and (3-bromopropyl)benzene (0.20 mmol, 30.4 μL) following Procedures C and F. The residue was purified by flash chromatography on silica gel eluting with 5% MeOH in CH_2_Cl_2_. Yield: 45%, yellow solid. ^1^H NMR (200 MHz, CDCl_3_—free base) δ 7.11–7.39 (m, 10H), 3.78–3.90 (m, 2H), 3.72 (s, 2H), 2.30–2.91 (m, 12H), 1.71–1.97 (m, 6H). ^13^C NMR (200 MHz, CDCl_3_—free base) δ 141.0, 139.3, 128.6, 128.4, 128.3, 126.3, 126.0, 65.9, 65.7, 57.9, 55.8, 53.6, 47.6, 37.7, 37.5, 34.6, 33.3, 28.8. Elem. Anal. for C_24_H_32_N_2_·H_2_C_2_O_4_, calcd: C, 71.21; H, 7.81; N, 6.39; found: C, 71.79; H, 7.87; N, 6.36.

##### *tert*-Butyl 7-Benzoyl-2,7-diazaspiro[3.5]nonane-2-carboxylate (**3a**)

The compound was prepared using *tert*-butyl 2,7-diazaspiro[3.5]nonane-2-carboxylate (50 mg, 0.22 mmol) and benzoyl chloride (58 μL, 0.44 mmol) following Procedure D. The residue was purified by flash chromatography on silica gel eluting with 2% MeOH in CH_2_Cl_2_. Yield: 75%, yellow oil. ^1^H NMR (200 MHz, CDCl_3_) δ 7.44–7.36 (m, 5H), 3.69 (m, 6H), 3.35 (br s, 2H), 1.76 (m, 4H) 1.44 (s, 9H).

##### *tert*-Butyl 7-(2-Phenylacetyl)-2,7-diazaspiro[3.5]nonane-2-carboxylate (**AB17**, **3b**)

The compound was prepared using *tert*-butyl 2,7-diazaspiro[3.5]nonane-2-carboxylate (50 mg, 0.22 mmol) and 2-phenylacetyl chloride (58 μL, 0.44 mmol). following Procedure D. The residue was purified by flash chromatography on silica gel eluting with 2% MeOH in CH_2_Cl_2_. Yield: 84%, yellow oil. ^1^H NMR (200 MHz, CDCl_3_) δ 7.18–7.41 (m, 5H), 3.74 (s, 2H), 3.50–3.69 (m, 6H), 3.30–3.40 (m, 2H), 1.64–1.77 (m, 2H), 1.47–1.57 (m, 2H), 1.43 (s, 9H).

##### (2-Phenethyl-2,7-diazaspiro[3.5]nonan-7-yl)(phenyl)methanone (**AB19**, **5a**)

The compound was prepared using **3b** (0.30 mmol, 95 mg) and (2-bromoethyl)benzene (0.50 mmol, 74 μL) following Procedures C and F. The residue was purified by flash chromatography on silica gel eluting with 0–2% MeOH in CH_2_Cl_2_. Yield: 27%, colorless oil. ^1^H NMR (500 MHz, CDCl_3_—free base) δ 7.34–7.43 (m, 5H), 7.26–7.31 (m, 2H), 7.18–7.23 (m, 3H), 3.66 (br. s., 2H), 3.32 (br. s., 2H), 3.16 (br. s., 4H), 2.77–2.85 (m, 2H), 2.68–2.75 (m, 2H), 1.80 (br. s., 4H). ^13^C NMR (200 MHz, CDCl_3_—free base) δ 170.5, 139.4, 136.1, 129.7, 128.8, 128.6, 126.9, 126.4, 64.0, 61.2, 53.6, 45.0, 39.5, 36.0, 34.8, 34.1. Elem. Anal. for C_22_H_26_N_2_O·H_2_C_2_O_4_, calcd: C, 79.00; H, 7.84; N, 8.38; found: C, 79.02; H, 7.85; N, 8.40.

##### 1-(2-Phenethyl-2,7-diazaspiro[3.5]nonan-7-yl)-2-phenylethan-1-one (**AB21**, **5b**)

The compound was prepared using **3a** (0.30 mmol, 95 mg) and (2-bromoethyl)benzene (0.50 mmol, 74 μL) following Procedures C and F. The residue was purified by flash chromatography on silica gel eluting with 0–2% MeOH in CH_2_Cl_2_. Yield: 27%, colorless oil. ^1^H NMR (500 MHz, CDCl_3_—free base) δ 6.75–7.82 (m, 10H), 3.71 (s, 2H), 3.48–3.56 (m, 2H), 3.29–3.37 (m, 2H), 3.20 (d, *J* = 7.8 Hz, 2H), 3.07–3.15 (m, 2H), 2.83 (t, *J* = 7.3 Hz, 2H), 2.69–2.77 (m, 2H), 1.65–1.76 (m, 2H), 1.60 (d, *J* = 4.9 Hz, 2H). ^13^C NMR (200 MHz, CDCl_3_—free base) δ 13C NMR (50 MHz, CHLOROFORM-d) d 169.5, 138.9, 135.2, 128.9, 128.8, 128.6, 126.9, 126.6, 63.9, 60.9, 43.5, 41.3, 39.1, 35.8, 35.7, 34.7, 33.8. Elem. Anal. for C_23_H_28_N_2_O·H_2_C_2_O_4_, calcd: C, 79.27; H, 8.10; N, 8.04; found: C, 79.28; H, 8.12; N, 8.05.

##### *tert*-Butyl Octahydro-1*H*-pyrrolo[3,4-*b*]pyridine-1-carboxylate (**6b**)

To a solution of (*S*,*S*)-2,8-diazabicyclo[4,3,0]nonane (7.37 mmol, 902 μL) in CH_2_Cl_2_ (30 mL), di-*tert*-butyl dicarbonate (5.89 mmol, 855 μL) in CH_2_Cl_2_ (30 mL) was added dropwise at 0 °C. The mixture was left to stir at rt on. The solvent was evaporated to dryness, and the obtained residue purified by flash chromatography on silica gel eluting with 10–40% MeOH in CH_2_Cl_2_. Yield: 64%, light pink solid. ^1^H NMR (200 MHz, CDCl_3_) δ 3.20–3.53 (m, 5H), 2.91–3.12 (m, 1H), 2.54–2.78 (m, 2H), 2.11–2.38 (m, 1H), 1.67 (br. s., 3H), 1.44 (s, 9H).

##### *tert*-Butyl 6-Phenyloctahydro-1*H*-pyrrolo[3,4-*b*]pyridine-1-carboxylate (**7a**)

The compound was prepared using iodobenzene (0.74 mmol, 83 μL) and **6b** (0.88 mmol, 200 mg) following Procedure A. The crude product was purified by column chromatography on silica gel with hexane/EtOAc (90:10 to 70:30) as the eluent to afford the desired product. Yield: 89%, brown oil. ^1^H NMR (200 MHz, CDCl_3_) δ 7.17–7.36 (m, 2H), 6.89–7.00 (m, 2H), 6.75–6.88 (m, 1H), 4.42 (d, *J* = 6.5 Hz, 1H), 3.29–3.50 (m, 4H), 3.12–3.29 (m, 1H), 2.81–3.02 (m, 1H), 2.21–2.43 (m, 1H), 1.51–1.94 (m, 4H), 1.44 (s, 9H).

##### *tert*-Butyl 6-Benzyloctahydro-1*H*-pyrrolo[3,4-*b*]pyridine-1-carboxylate (**7b**)

The compound was prepared using **6b** (0.88 mmol, 200 mg) and benzylbromide (1.70 mmol, 201 μL) following Procedure B. The residue was purified by flash chromatography on silica gel eluting with 10–20% MeOH in EtOAc to give the title compound. Yield: 90%, yellow oil. ^1^H NMR (200 MHz, CDCl_3_) δ 7.19–7.40 (m, 5H), 5.25–5.33 (m, 1H), 3.50–3.83 (m, 2H), 3.15–3.45 (m, 4H), 3.06 (br. s., 1H), 2.59 (br. s., 1H), 2.27 (br. s., 2H), 1.51–1.91 (m, 3H), 1.47 (s, 9H).

##### *tert*-Butyl 6-Phenethyloctahydro-1*H*-pyrrolo[3,4-*b*]pyridine-1-carboxylate (**7c**)

The compound was prepared using **6b** (0.88 mmol, 200 mg) and (2-bromoethyl)benzene (1.70 mmol, 229 μL) following Procedure B. The residue was purified by flash chromatography on silica gel eluting with 10–20% MeOH in EtOAc to give the title compound. Yield: 97%, yellow oil. ^1^H NMR (200 MHz, CDCl_3_) δ 7.06–7.41 (m, 5H), 3.12–3.62 (m, 5H), 2.36–2.93 (m, 6H), 2.24 (br. s., 1H), 1.53–1.95 (m, 4H), 1.46 (s, 9H).

##### 1-Benzyl-6-phenyloctahydro-1*H*-pyrrolo[3,4-*b*]pyridine (**AD260**, **8a**)

The compound was prepared using **7a** (0.35 mmol, 150 mg) and benzylbromide (0.70 mmol, 94 μL) following Procedures C and F. The obtained residue was purified on silica gel chromatography with 0–20% MeOH in EtOAc. Yield: 35%, orange oil. ^1^H NMR (500 MHz, CDCl_3_—free base) δ 7.17–7.44 (m, 7H), 6.87–7.02 (m, 3H), 3.99–4.10 (m, 2H), 3.94 (br. s., 1H), 3.44 (d, *J* = 6.8 Hz, 2H), 3.12–3.28 (m, 2H), 2.81–3.00 (m, 2H), 2.68 (br. s., 1H), 1.81–1.92 (m, 1H), 1.68–1.76 (m, 2H), 1.55–1.66 (m, 1H). ^13^C NMR (200 MHz, CDCl_3_—free base) δ 150.2, 131.5, 130.1, 129.5, 129.2, 129.0, 122.4, 120.4, 61.0, 57.3, 55.2, 54.6, 37.2, 22.9, 22.0 Elem. Anal. for C_20_H_24_N_2_·H_2_C_2_O_4_, calcd: C, 82.15; H, 8.27; N, 9.58; found: C, 82.17; H, 8.26; N, 9.60.

##### 1-Phenethyl-6-phenyloctahydro-1*H*-pyrrolo[3,4-*b*]pyridine (**AB13**, **8b**)

The compound was prepared using **7a** (0.35 mmol, 100 mg) and (2-bromoethyl)benzene (0.70 mmol, 94 μL) following Procedures C and F. The obtained residue was purified on silica gel chromatography with 0–20% MeOH in EtOAc. Yield: 43%, white solid. ^1^H NMR (200 MHz, CDCl_3_—free base) δ 7.12–7.37 (m, 7H), 6.91 (d, *J* = 8.0 Hz, 2H), 6.78 (t, *J* = 8.0 Hz, 1H), 4.37 (q, *J* = 7.7 Hz, 1H), 3.27–3.46 (m, 1H), 2.86–3.11 (m, 3H), 2.68–2.82 (m, 4H), 2.54–2.67 (m, 2H), 2.39 (br. s., 1H), 1.50–2.00 (m, 4H). ^13^C NMR (200 MHz, CDCl_3_—free base) δ 150.5, 140.3, 129.1, 128.7, 128.3, 126.0, 118.1, 115.0, 59.0, 58.7, 56.4, 53.0, 44.5, 36.3, 35.4, 26.1, 23.5. Elem. Anal. for C_21_H_26_N_2_·H_2_C_2_O_4_, calcd: C, 69.68; H, 7.12; N, 7.07; found: C, 70.09; H, 7.17; N, 7.05.

##### 6-Benzyl-1-phenethyloctahydro-1*H*-pyrrolo[3,4-*b*]pyridine (**AB9**, **8c**)

The compound was prepared using **7b** (0.38 mmol, 120 mg) and (2-bromoethyl)benzene (0.75 mmol, 102 μL) following Procedures C and F. The obtained residue was purified on silica gel chromatography with 0–20% MeOH in CH_2_Cl_2_. Yield: 30%, yellow solid. ^1^H NMR (200 MHz, CDCl_3_—free base) δ 7.11–7.43 (m, 10H), 3.57–3.87 (m, 3H), 3.11–3.45 (m, 7H), 2.80–3.02 (m, 2H), 2.68 (d, *J* = 4.9 Hz, 1H), 1.92–2.18 (m, 1H), 1.47–1.87 (m, 3H). ^13^C NMR (200 MHz, CDCl_3_—free base) δ 137.6, 136.1, 128.9, 128.6, 128.3, 127.4, 127.2, 61.9, 59.3, 59.2, 57.3, 55.1, 51.9, 37.8, 32.2, 21.8, 20.7. Elem. Anal. for C_22_H_28_N_2_·H_2_C_2_O_4_, calcd: C, 70.22; H, 7.37; N, 6.82; found: C, 70.53; H, 7.38; N, 6.78.

##### 1,6-Diphenethyloctahydro-1*H*-pyrrolo[3,4-*b*]pyridine (**AB7**, **8d**)

The compound was prepared using **7b** (0.49 mmol, 160 mg) and (3-bromopropyl)benzene (0.98 mmol, 133 μL) following Procedures C and F. The obtained residue was purified on silica gel chromatography with 10–20% MeOH in CH_2_Cl_2_. Yield: 49%, orange solid. ^1^H NMR (200 MHz, CDCl_3_—free base) δ 6.83–7.46 (m, 10H), 4.88 (s, 1H), 4.18 (dd, *J* = 3.4, 12.6 Hz, 1H), 3.83–4.05 (m, 1H), 3.41–3.70 (m, 4H), 3.00–3.38 (m, 4H), 2.95 (br. s., 1H), 2.53–2.89 (m, 4H), 2.26–2.46 (m, 1H), 1.87–2.10 (m, 1H), 1.63 (br. s., 2H). ^13^C NMR (200 MHz, CDCl_3_—free base) δ 140.3, 135.2, 129.2, 129.0, 128.7, 128.3, 127.6, 127.4, 69.1, 65.9, 64.9, 63.2, 62.4, 56.3, 51.3, 36.5, 32.6, 30.3, 29.7. Elem. Anal. for C_23_H_30_N_2_·H_2_C_2_O_4_, calcd: C, 70.73; H, 7.60; N, 6.60; found: C, 70.97; H, 7.66; N, 6.59.

##### 6-Phenyl-1-(3-phenylpropyl)octahydro-1*H*-pyrrolo[3,4-*b*]pyridine (**AB14**, **8e**)

The compound was prepared using **7a** (0.35 mmol, 100 mg) and (3-bromopropyl)benzene (0.52 mmol, 79 μL) following Procedures C and F. The obtained residue was purified on silica gel chromatography with 0–20% MeOH in EtOAc. Yield: 97%, white solid. ^1^H NMR (200 MHz, CDCl_3_—free base) δ 7.13–7.37 (m, 7H), 6.93 (d, *J* = 8.0 Hz, 2H), 6.80 (t, *J* = 7.2 Hz, 1H), 4.38 (q, *J* = 7.7 Hz, 1H), 3.32–3.47 (m, 1H), 2.79–3.08 (m, 3H), 2.28–2.72 (m, 7H), 1.54–2.00 (m, 5H). ^13^C NMR (200 MHz, CDCl_3_—free base) δ 150.5, 142.2, 129.0, 128.4, 128.2, 125.6, 118.0, 114.9, 58.7, 56.5, 56.3, 53.1, 44.5, 36.3, 33.6, 30.4, 26.2, 23.5. Elem. Anal. for C_22_H_28_N_2_·H_2_C_2_O_4_, calcd: C, 70.22; H, 7.37; N, 6.82; found: C, 70.53; H, 7.42; N, 6.79.

##### 6-Benzyl-1-(3-phenylpropyl)octahydro-1*H*-pyrrolo[3,4-*b*]pyridine (**AB10**, **8f**)

The compound was prepared using **7c** (0.38 mmol, 120 mg) and (3-bromopropyl)benzene (0.56 mmol, 85 μL) following Procedures C and F. The obtained residue was purified on silica gel chromatography with 10–20% MeOH in CH_2_Cl_2_. Yield: 16%, yellow solid. ^1^H NMR (200 MHz, CDCl_3_—free base) δ 7.08–7.51 (m, 10H), 3.75 (d, *J* = 13.8 Hz, 2H), 3.38–3.62 (m, 1H), 2.47–3.30 (m, 9H), 1.90–2.31 (m, 3H), 1.42–1.87 (m, 3H). ^13^C NMR (200 MHz, CDCl_3_—free base) δ 140.9, 135.8, 129.8, 129.6, 129.3, 129.0, 128.3, 128.0, 69.7, 66.6, 65.5, 63.8, 63.0, 56.9, 52.0, 37.1, 33.2, 31.0, 30.3 Elem. Anal. for C_23_H_30_N_2_·H_2_C_2_O_4_, calcd: C, 70.73; H, 7.60; N, 6.60; found: C, 71.37; H, 7.65; N, 6.58.

##### 6-Phenethyl-1-(3-phenylpropyl)octahydro-1*H*-pyrrolo[3,4-*b*]pyridine (**AB8**, **8g**)

The compound was prepared using **7c** (0.49 mmol, 160 mg) and (3-bromopropyl)benzene (0.74 mmol, 112 μL) following Procedures C and F. The obtained residue was purified on silica gel chromatography with 10–20% MeOH in CH_2_Cl_2_. Yield: 73%, orange solid. ^1^H NMR (200 MHz, CDCl_3_—free base) δ 7.11–7.43 (m, 10H), 4.03–4.26 (m, 1H), 3.72–3.97 (m, 1H), 3.22–3.61 (m, 3H), 2.88–3.19 (m, 3H), 2.51–2.86 (m, 4H), 2.45 (t, *J* = 7.6 Hz, 2H), 2.28 (d, *J* = 7.2 Hz, 4H), 1.84–2.08 (m, 2H), 1.41–1.83 (m, 4H). ^13^C NMR (200 MHz, CDCl_3_—free base) δ 140.1, 139.8, 128.8, 128.6, 128.4, 128.2, 126.8, 126.5, 68.5, 65.3, 62.2, 61.6, 56.0, 51.2, 36.5, 32.5, 32.0, 31.7, 25.1, 24.9. Elem. Anal. for C_24_H_32_N_2_·H_2_C_2_O_4_, calcd: C, 71.21; H, 7.81; N, 6.39; found: C, 71.79; H, 7.87; N, 6.35.

### Radioligand Binding Assays

#### S1R and S2R Binding Affinity

Liver homogenates for S1R and S2R receptor binding assays were prepared from male Sprague-Dawley rats as previously reported.^46,53^*In vitro* S1R ligand binding assays were carried out in Tris buffer (50 mM, pH 8), using [^3^H](+)-pentazocine (2 nM) in a final volume of 0.5 mL with increasing concentrations of test compounds. The *K*_d_ value of [^3^H](+)-pentazocine was 2.9 nM. Unlabeled (+)-pentazocine (10 μM) was used to measure nonspecific binding.^46^*In vitro* S2R ligand binding assays were carried out in Tris buffer (50 mM, pH 8.0), using [^3^H]DTG (2 nM) in the presence of (+)-pentazocine (5 μM) as S1R masking agent and increasing concentrations of test compounds in a final volume of 0.5 mL. The *K*_d_ value of [^3^H]DTG was 17.9 nM. Nonspecific binding was evaluated with unlabeled DTG (10 μM).^53^

#### S1R Functional Assay

The functionality of compounds **4b**, **5b**, and **8f** on S1R was determined using the same radiolabeled binding assay for the S1R in the presence of phenytoin. Binding experiments were carried out by incubating 200 μL of membrane preparation with 50 μL of 20 nM [^3^H](+)-pentazocine (26.9 Ci/mmol, PerkinElmer), 50 μL of cold ligand or its solvent, and 20 μL of 25 mM phenytoin (Merck Life Science S.r.l.) or its solvent (0.3 M NaOH) for 120 min at 37 °C. The final volume was 0.5 mL. Unlabeled (+)-pentazocine (10 μM) was used to measure nonspecific binding. If the *K*_i_ ratio without/with phenytoin is >1, the test compound acts as S1R agonists. On the contrary, if the *K*_i_ ratio without/with phenytoin is ≤1, the compound is considered S1R antagonists.^52^

#### Data Analysis

The *K*_i_-values were calculated with the program GraphPad Prism 7.0 (GraphPad Software, San Diego, California). The *K*_i_-values are given as mean value ± SD from at least two independent experiments performed in duplicate.

### Molecular Modeling

#### Active and Decoy Compounds

The active compounds were extrapolated from the ChEMBL database.^54^ We considered for the S1R, 15 compounds with a range of *K*_i_ of 0.005–5 nM and 15 compounds with a *K*_i_ range of 0.12–8.2 nM on the S2R. For the generation of the decoys set, we used the DUDE-Z online server generating two datasets of 750 and 850 decoys for S1R and S2R, respectively.^55^

#### Ligand Preparation

All of the compounds were prepared by means of the LigPrep tool. Hydrogens were added, salts were removed, and ionization states were calculated using Epik at pH 7.4 and OPLS_2005 as force field (LigPrep, Schrödinger, LLC, New York, NY, 2018).

#### Receptor Preparation and Validation

Starting from the crystal structure of the human S1R bound to PD144418 deposited in the Protein Data Bank with the PDB code 5HK1, our molecular modeling analysis was carried out.^2^ The receptor structure was prepared by means of Protein Preparation Wizard tool using OLPS_2005 as force field. Residual crystallographic buffer components were removed, missing side chains were built using the Prime module, hydrogen atoms were added, and side chain protonation states at pH 7.4 were assigned (Protein Preparation Wizard, Schrödinger, LLC, New York, NY, 2018). We submitted the receptor structure to 100 ns of molecular dynamics using Desmond package v. 3.8. (Desmond Molecular Dynamics System, D.E. Shaw Re-search, New York, NY, 2018 and Maestro-Desmond Interoperability Tools, Schrödinger, New York, NY, 2018). The structure was embedded in a fully hydrated palmitoyl-oleyl-phosphatidylcholine (POPC) bilayer, and the system was immersed in an orthorhombic box of TIP4P water molecules, extending at least 10 Å from the protein, and counter ions were added to neutralize the system charge. The system temperature was set at 300 K, and the NPT ensemble was selected. The resulting trajectory was clustered with respect to the root mean square deviation (RMSD), getting four cluster representatives. These structures were submitted to 10,000 iterations of energy minimization using the MacroModel tool and OPLS-2005 as the force field (MacroModel, Schrödinger, LLC, New York, NY, 2018), obtaining four additional structures for further molecular docking studies.

Regarding the S2R, we used the crystal structure of the bovine S2R bound to compound Z1241145220 deposited in the Protein Data Bank with the PDB code 7M95.^56^ In order to generate the full-length model of the human receptor, we used the UniProtKB—Q5BJF2 (SGMR2_HUMAN) sequence obtained from the UniProt. The receptor structure was prepared by means of Protein Preparation Wizard tool using OLPS_2005 as force field. Residual crystallographic buffer components were removed, missing side chains were built using the Prime module, hydrogen atoms were added, and side chain protonation states at pH 7.4 were assigned (Protein Preparation Wizard, Schrödinger, LLC, New York, NY, 2018). For the molecular dynamics simulations, the same protocol described for the S1R was used. The trajectory clusterization produced six cluster representatives, which were minimized thus obtaining 12 structures.

#### Docking Protocol Validation

According to the enrichment factor, AUC, and Receiver Operating Characteristic (ROC) analyses, we chose the S1R and the S2R structures associated with the highest AUC and ROC values (S1R AUC value: 0.76 and ROC value: 0.77; S2R AUC value: 0.89 and ROC value: 0.89) for the further docking studies.

#### Docking Studies

Molecular docking was carried out using Glide v. 6.7 Standard Precision (SP) protocol, and 10 poses per ligand were generated (Glide, Schrödinger, LLC, New York, NY, 2018).

### *In Vivo* Assays

#### Experimental Animals

Experiments were performed in female CD1 mice (Charles River, Barcelona, Spain) weighing 25–30 g. The mice were acclimated in our animal facilities for at least 1 week before testing and housed in a room under controlled environmental conditions: 12/12 h day/night cycle, air replacement every 20 min, and regulated temperature (22 ± 2 °C). The animals were fed a standard laboratory diet (Harlan Teklad Research Diet, Madison, Wisconsin) and tap water *ad libitum* until the beginning of the experiments. Testing was always performed from 9.00 to 15.00 h and randomly throughout the oestrous cycle. Animal care was made in accordance with institutional (Research Ethics Committee of the University of Granada, Spain), regional (Junta de Andalucía, Spain), and international standards (European Communities Council Directive 2010/63).

#### Drugs and Drug Administration

As selective S1R drugs, we used the S1R antagonist BD-1063 (1-[2-(3,4-dichlorophenyl)ethyl]-4-methylpiperazine dihydrochloride) and the S1R agonist PRE-084 (2-(4-morpholinethyl)-1-phenyl cyclohexane carboxylate hydrochloride) (both provided by Tocris Cookson, Bristol, U.K.).^57^ The standard or experimental compounds were dissolved in 5% DMSO (Merck KGaA, Darmstadt, Germany) in physiological sterile saline (0.9% NaCl). They were prepared immediately before the start of the experiments and injected s.c. in a volume of 5 mL/kg into the interscapular area.

To test for the antiallodynic effects of BD-1063, **4b**, **5b**, or **8f**, they were administered 30 min before the injection of capsaicin, used as the chemical algogen. To test the effects of PRE-084 or **4b** on the antiallodynia induced by BD-1063, **8f**, or **5b**, the former were administered 5 min before the latter. When the effect of the association of several drugs was assessed, each injection was performed in different areas of the interscapular zone to avoid mixture of the drug solutions and any physicochemical interaction between them.

Capsaicin (Sigma-Aldrich Química S.A.) was dissolved in 1% DMSO in physiological sterile saline to a concentration of 0.05 μg/μL (i.e., 1 μg per mouse). Capsaicin solution was injected intraplantarly (i.pl.) into the right hind paw proximate to the heel, in a volume of 20 μL using a 1710 TLL Hamilton microsyringe (Teknokroma, Barcelona, Spain) with a 30^1/2^-gauge needle. Control animals were injected with the same volume of the vehicle of capsaicin.

#### Evaluation of Capsaicin-Induced Secondary Mechanical Hypersensitivity

Animals were placed for 2 h in individual black-walled test compartments, which were situated on an elevated mesh-bottom platform with a 0.5-cm^2^ grid to provide access to the ventral surface of the hind paws. Punctate mechanical stimulation was applied with a Dynamic Plantar Aesthesiometer (Ugo Basile, Varese, Italy) 15 min after capsaicin or saline administration (i.e., 45 min after the injection of the experimental drug). Briefly, a nonflexible filament (0.5 mm diameter) was electronically driven into the ventral side of the right hind paw (which was previously injected with capsaicin or vehicle) at least 5 mm away from the site of the injection toward the fingers. The intensity of the stimulation was fixed at 0.5 g force, as described previously.^49,50^ When a paw withdrawal response occurred, the stimulus was automatically terminated and the response latency was recorded. The filament was applied three times at intervals of 0.5 min, and the mean value of the three trials was considered the withdrawal latency time of the animal.

## Supporting Information Available

^1^H NMR and ^13^C NMR spectra of final compounds (PDF)
