# Supplementary material for: Synthesis, Computational Insights, and Evaluation of Novel Sigma Receptors Ligands
Source: ACS Chem Neurosci. 2023 May 8;14(10):1845–58. doi: 10.1021/acschemneuro.3c00074 (PMC10197133; doi:10.1021/acschemneuro.3c00074)

Supporting Information

Synthesis, computational insights, and evaluation of novel sigma receptors ligands

Maria Dichiara,[1] Francesca Alessandra Ambrosio,[2] Carla Barbaraci,[1] Rafael González-Cano,[3] Giosuè Costa,[4,5] Carmela Parenti,[1] Agostino Marrazzo,[1] Lorella Pasquinucci,[1] Enrique J. Cobos,[3] Stefano Alcaro,[4,5,*] Emanuele Amata[1,*]

[1]Dipartimento di Scienze del Farmaco e della Salute, Università degli Studi di Catania, Viale Andrea Doria 6, 95125, Catania, Italy;

[2]Dipartimento di Medicina Sperimentale e Clinica, Università degli Studi "Magna Græcia" di Catanzaro, Campus "S. Venuta", Viale Europa, 88100, Catanzaro, Italy;

[3]Departamento de Farmacología e Instituto de Neurociencias, Facultad de Medicina, Universitad de Granada e Instituto de Investigación Biosanitaria de Granada ibs.GRANADA, Avenida de la Investigación, 11 18016 Granada, Spain;

[4]Dipartimento di Scienze della Salute, Università "Magna Græcia" di Catanzaro, Campus "S. Venuta", 88100, Catanzaro, Italy;

[5]Net4Science Academic Spin-Off, Università "Magna Græcia" di Catanzaro, Campus "S. Venuta", 88100, Catanzaro, Italy.

Corresponding Author

*Emanuele Amata: phone, (+39) 0957384102; email, eamata@unict.it

*Stefano Alcaro: phone, (+39) 09613694198; email, alcaro@unicz.it

^1H and ^{13}C NMR-spectra in $CDCl_3$

2-Phenethyl-7-phenyl-2,7-diazaspiro[3.5]nonane (**AD172**, **4a**)

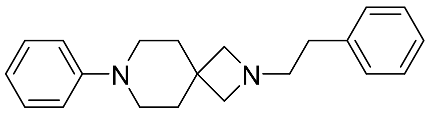

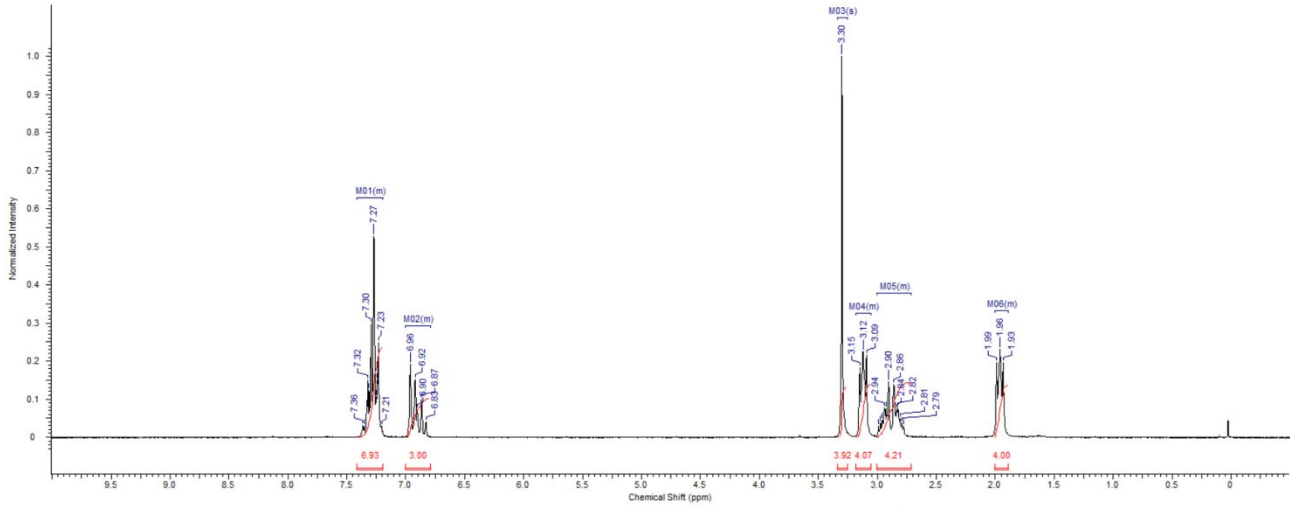

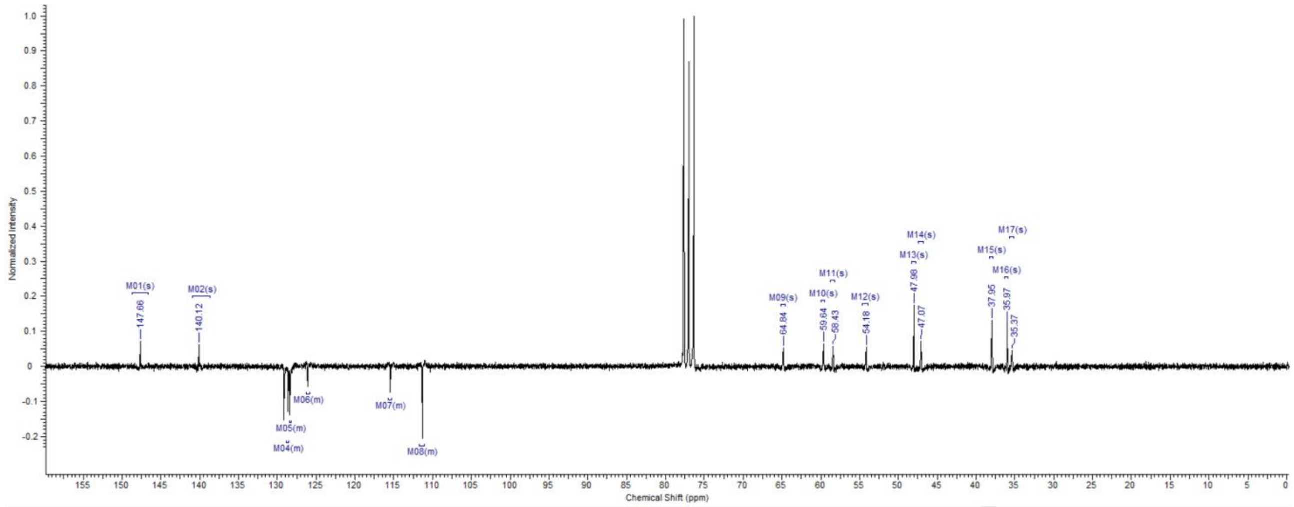

7-Benzyl-2-phenethyl-2,7-diazaspiro[3.5]nonane (**AD186**, **4b**)

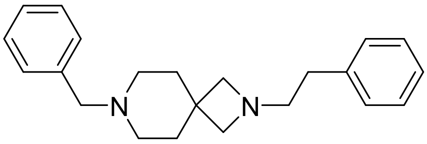

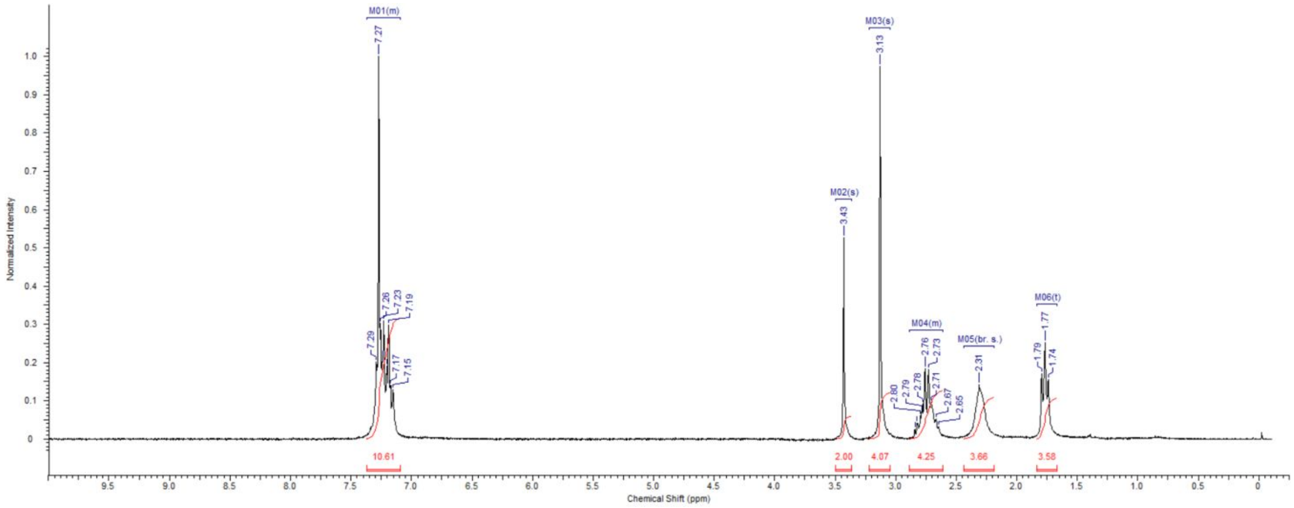

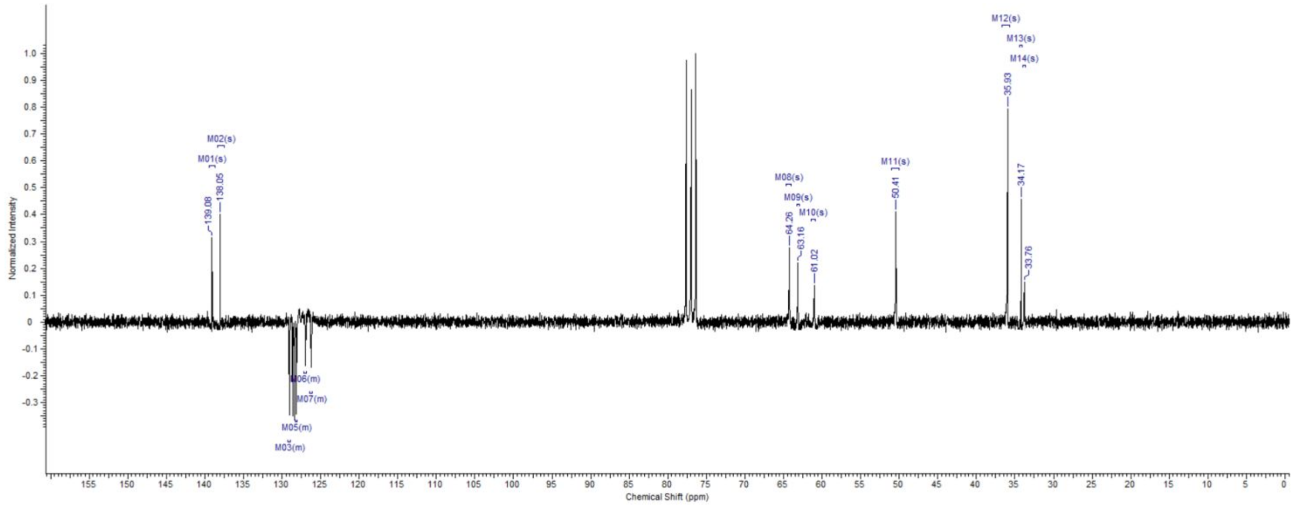

2,7-Diphenethyl-2,7-diazaspiro[3.5]nonane (**AD187**, **4c**)

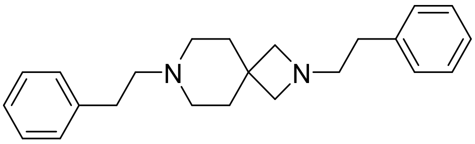

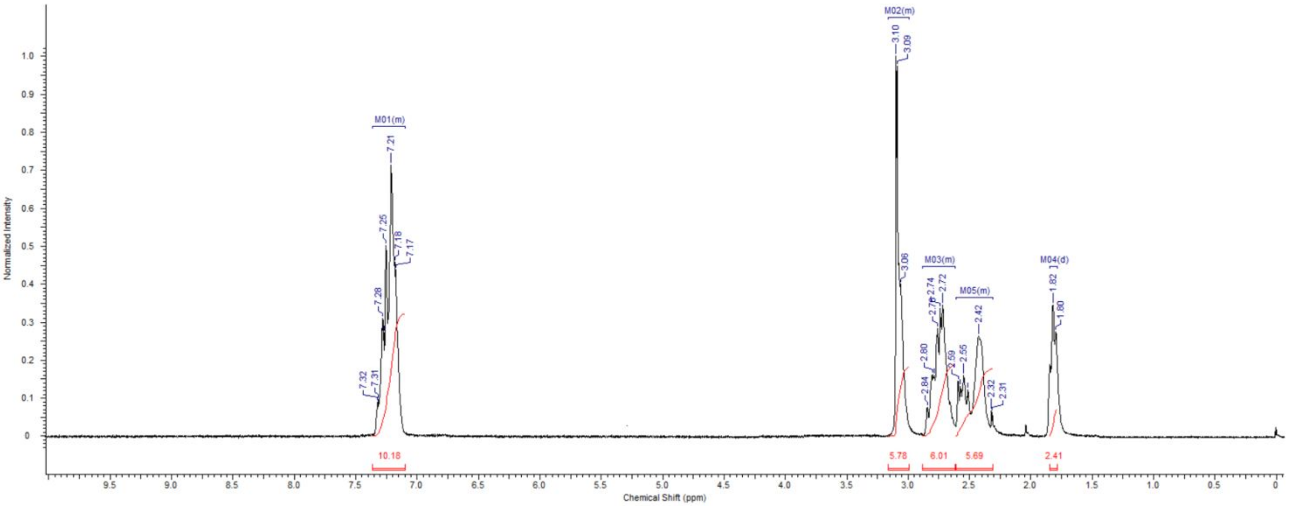

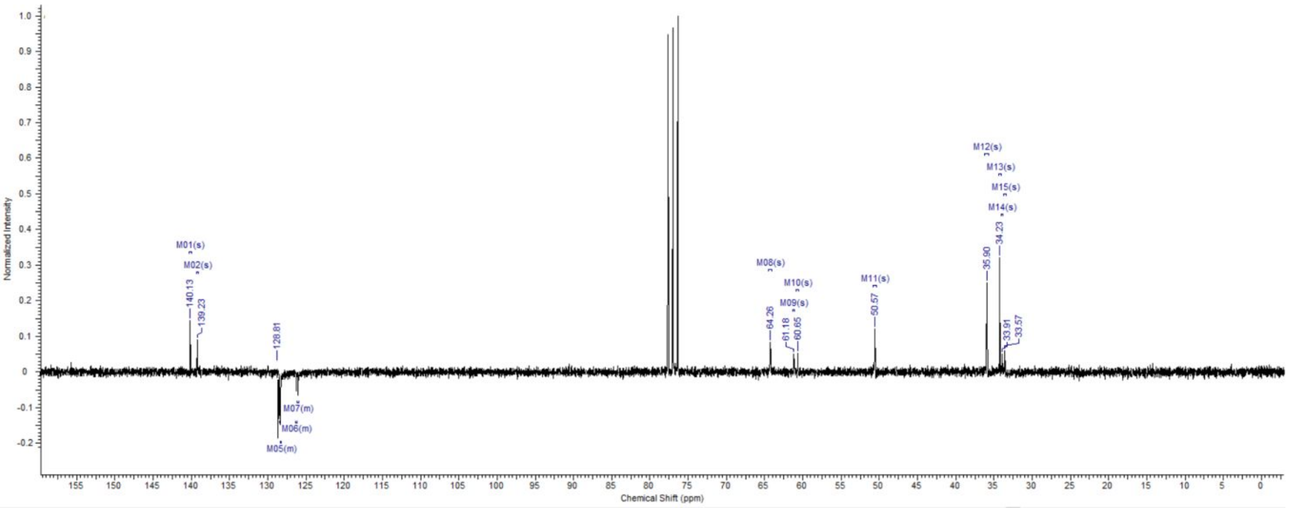

7-Phenyl-2-(3-phenylpropyl)-2,7-diazaspiro[3.5]nonane (**AD173**, **4d**)

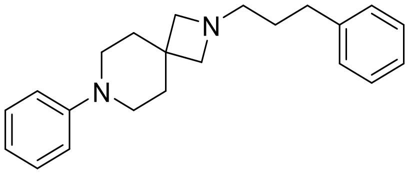

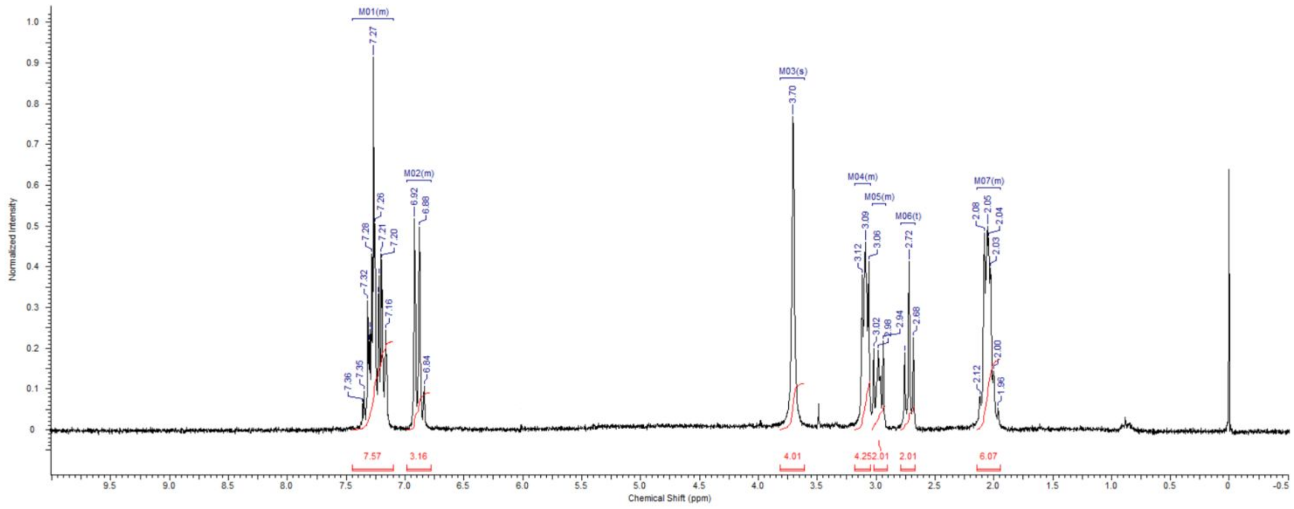

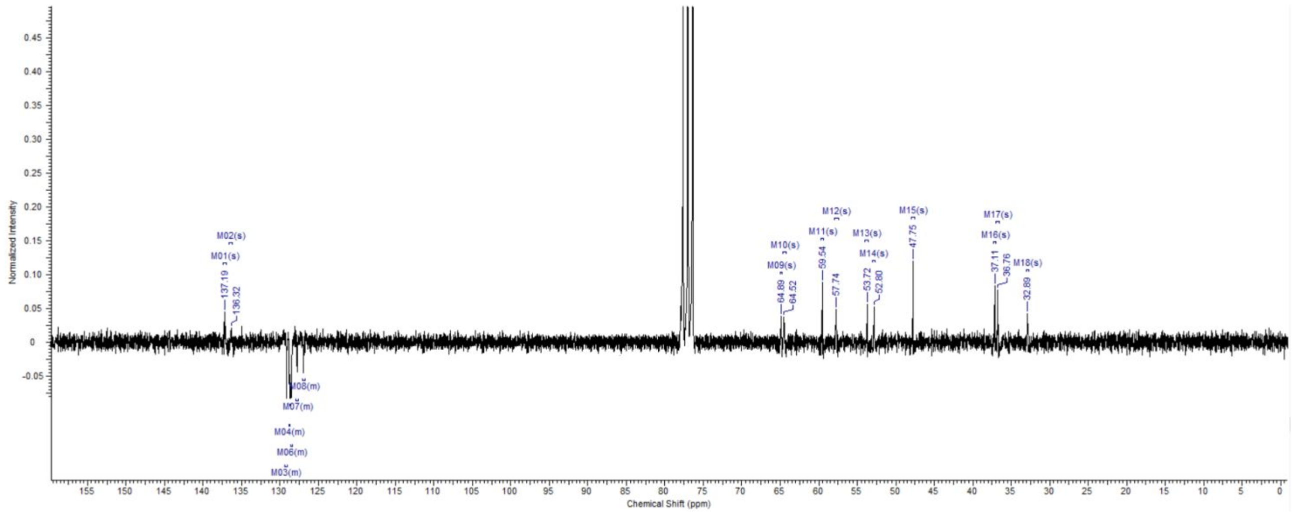

7-Benzyl-2-(3-phenylpropyl)-2,7-diazaspiro[3.5]nonane (**AD195**, **4e**)

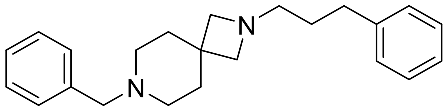

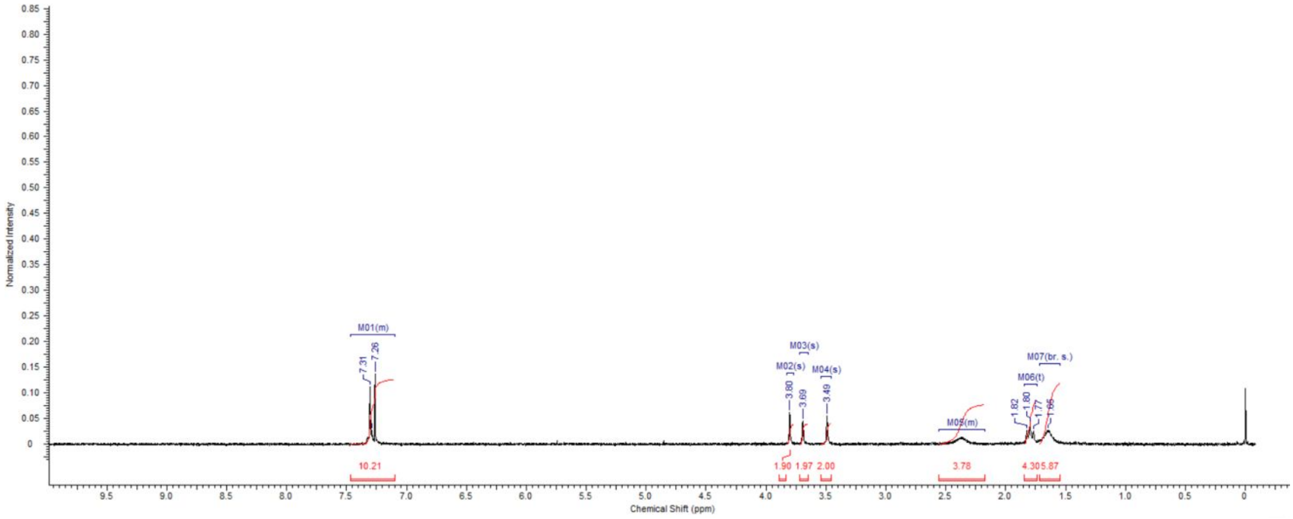

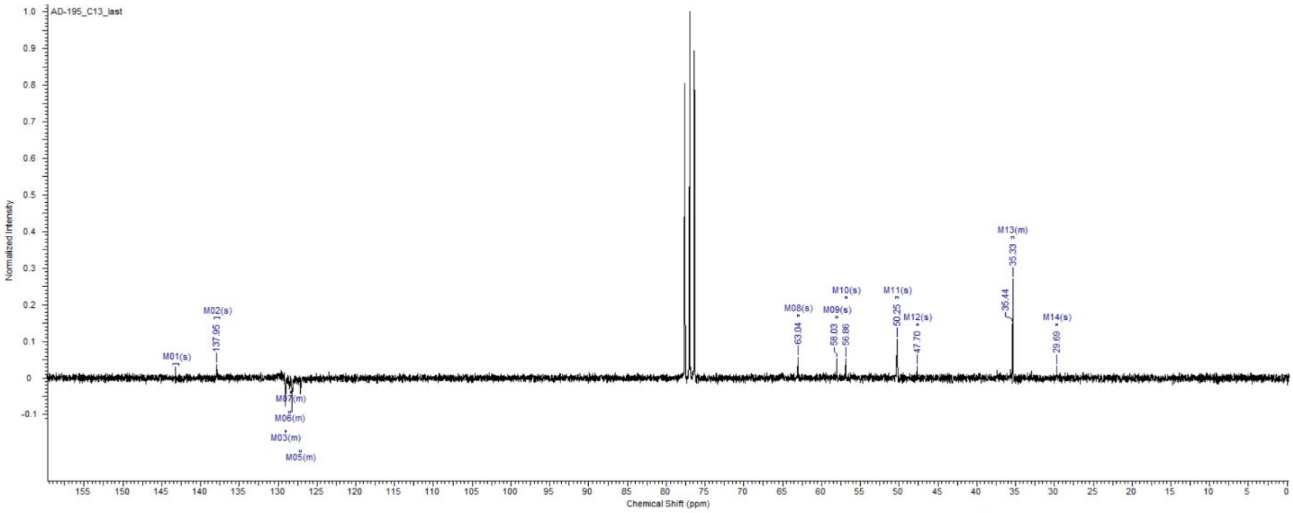

7-Phenethyl-2-(3-phenylpropyl)-2,7-diazaspiro[3.5]nonane (**AD197**, **4f**)

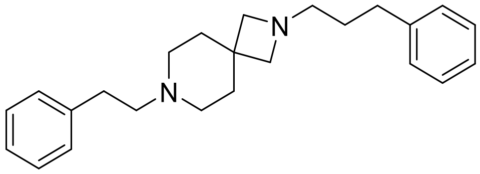

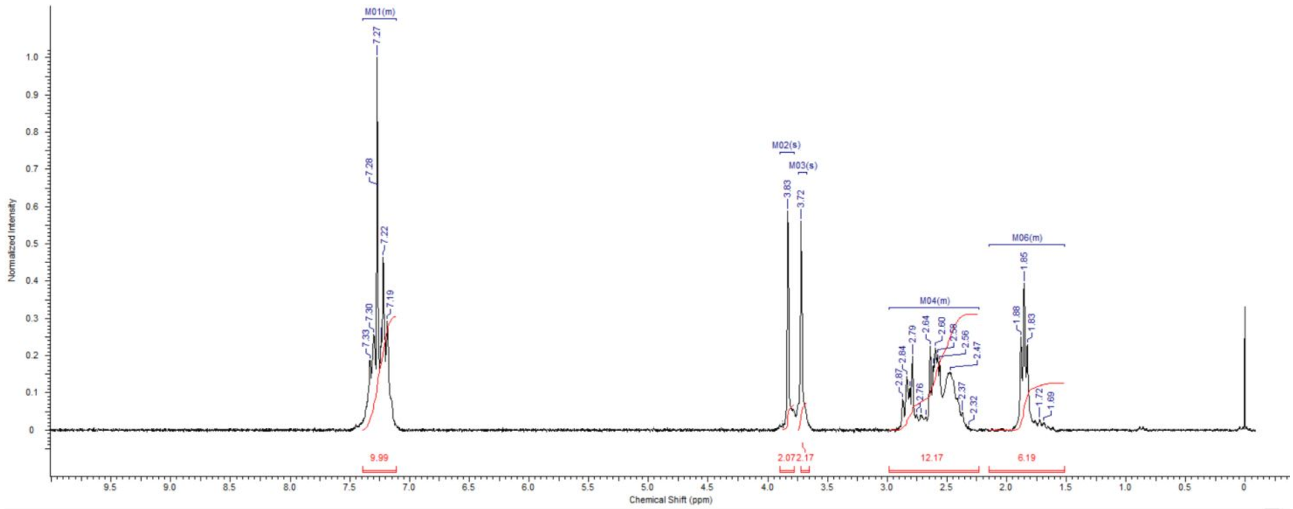

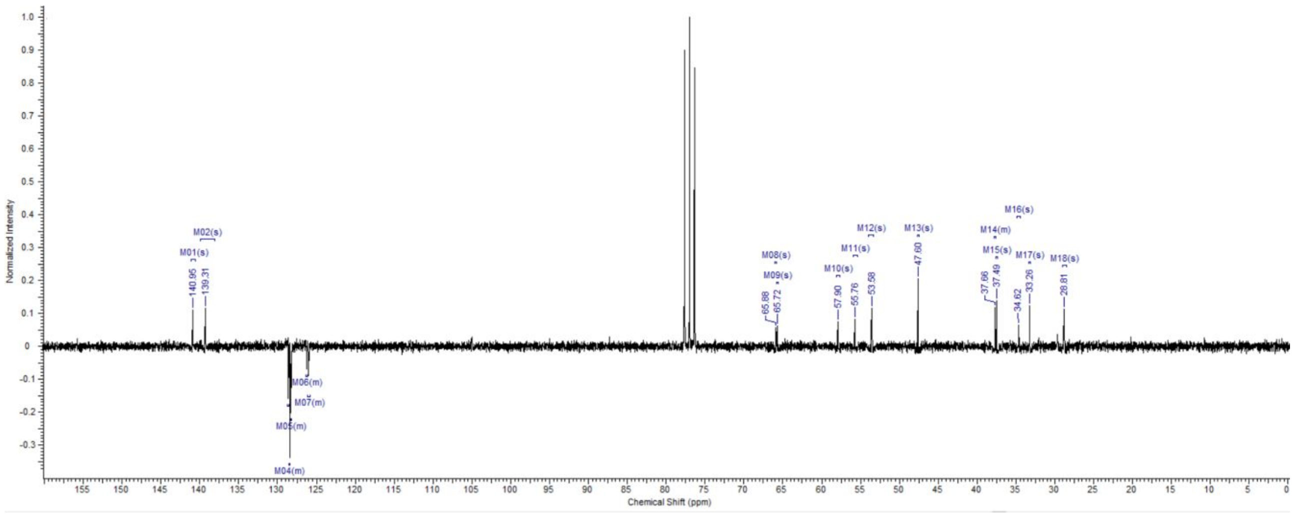

(2-Phenethyl-2,7-diazaspiro[3.5]nonan-7-yl)(phenyl)methanone (**AB19**, **5a**)

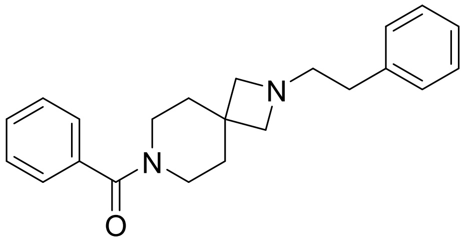

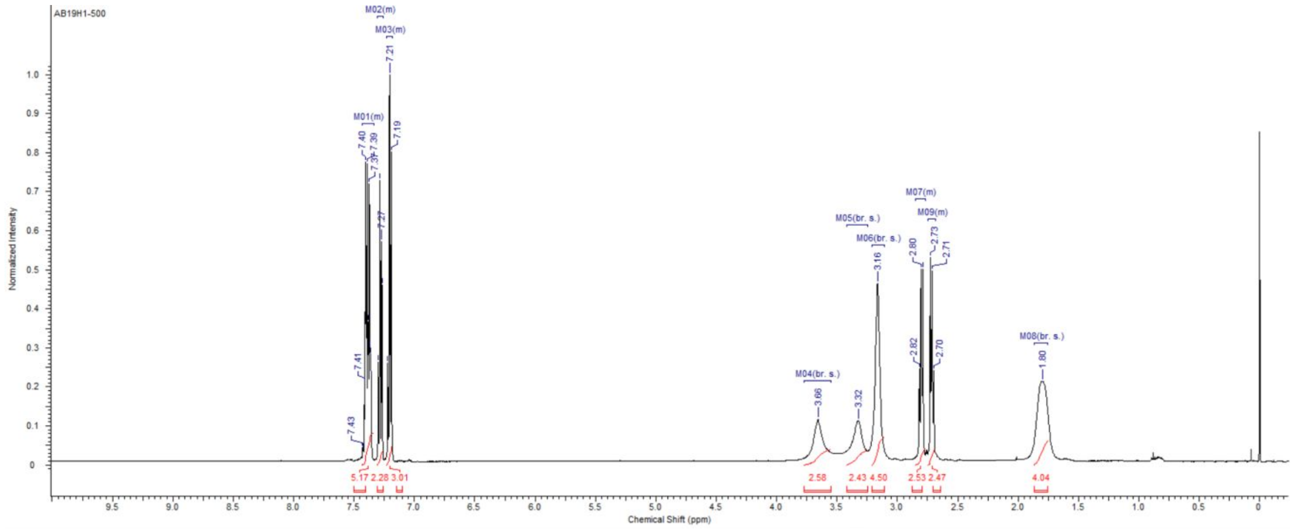

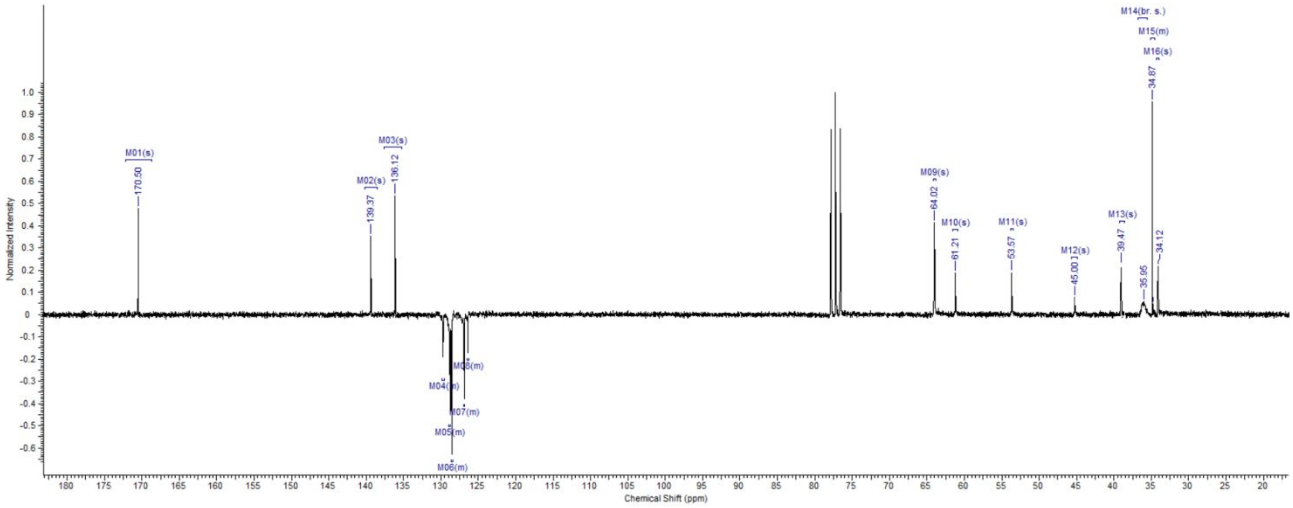

1-(2-Phenethyl-2,7-diazaspiro[3.5]nonan-7-yl)-2-phenylethan-1-one (**AB21**, **5b**)

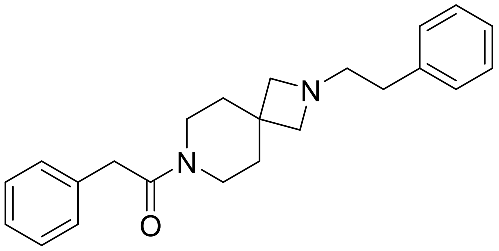

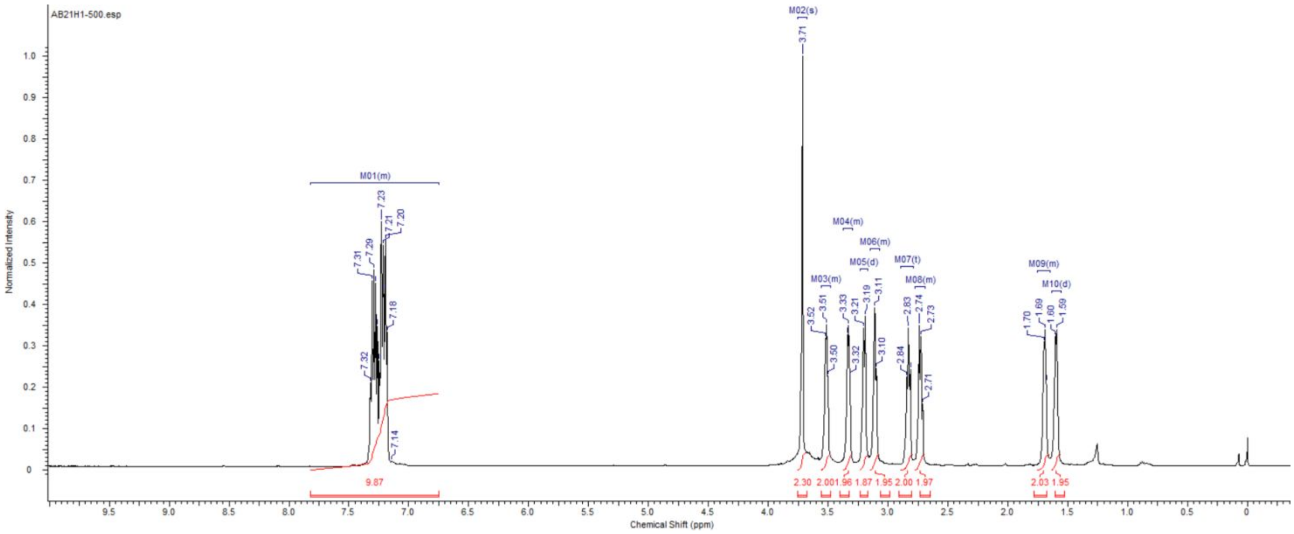

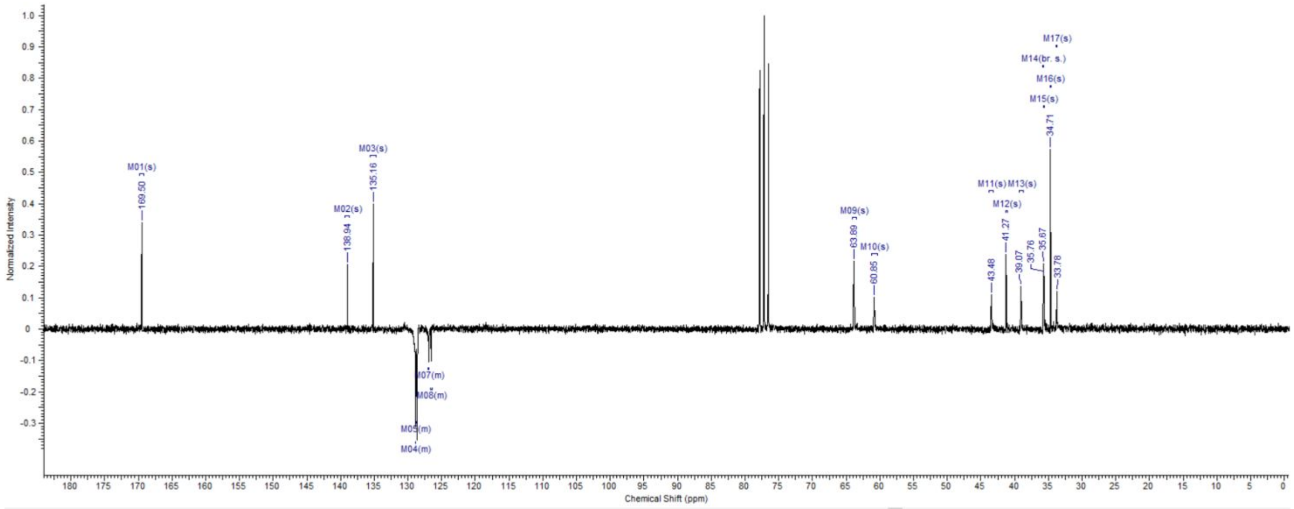

1-Benzyl-6-phenyloctahydro-1*H*-pyrrolo[3,4-b]pyridine (**AD260**, **8a**)

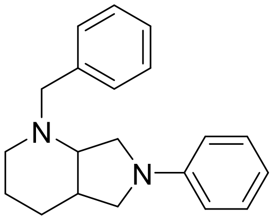

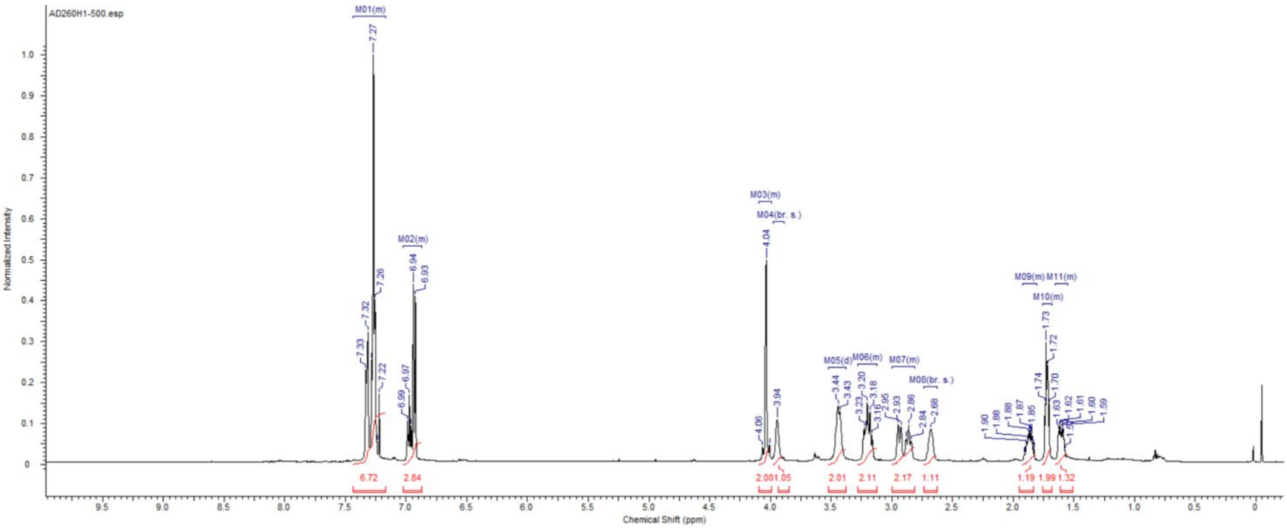

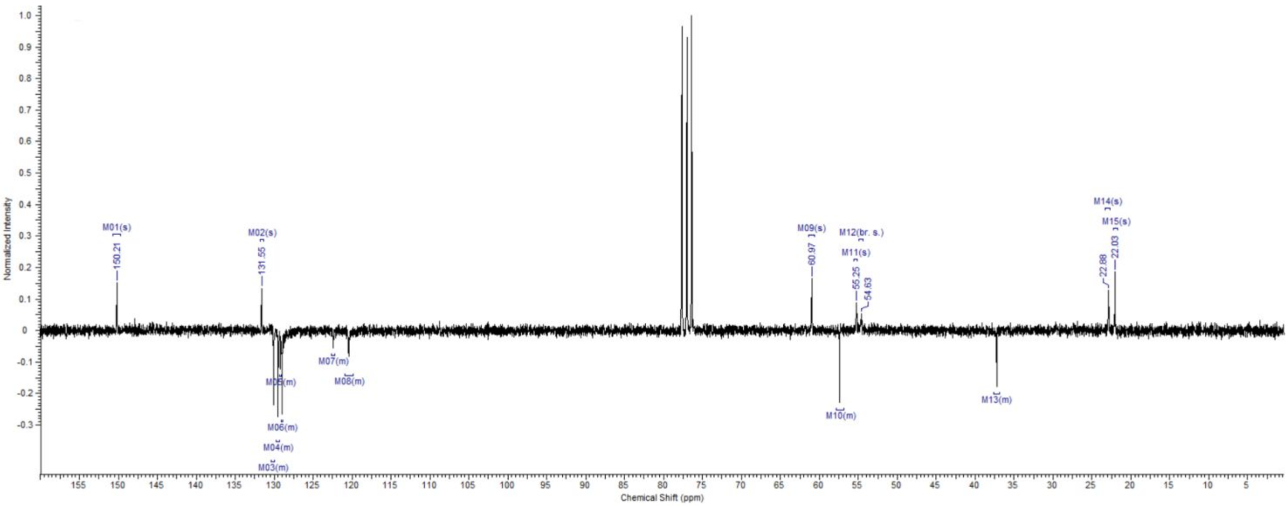

1-Phenethyl-6-phenyloctahydro-1*H*-pyrrolo[3,4-*b*]pyridine (**AB13**, **8b**)

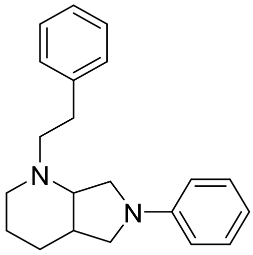

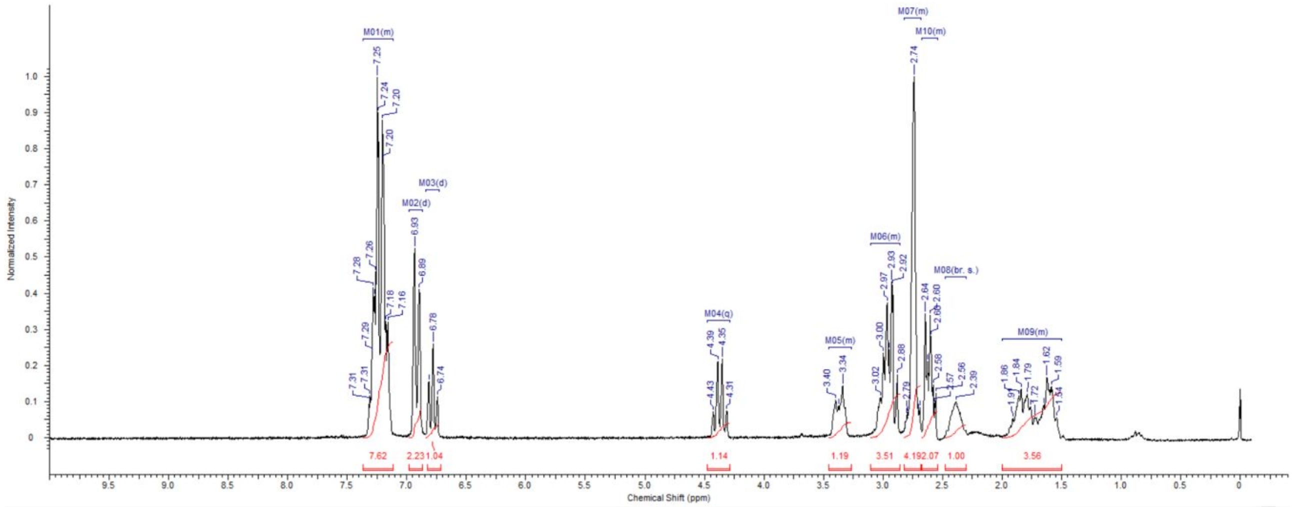

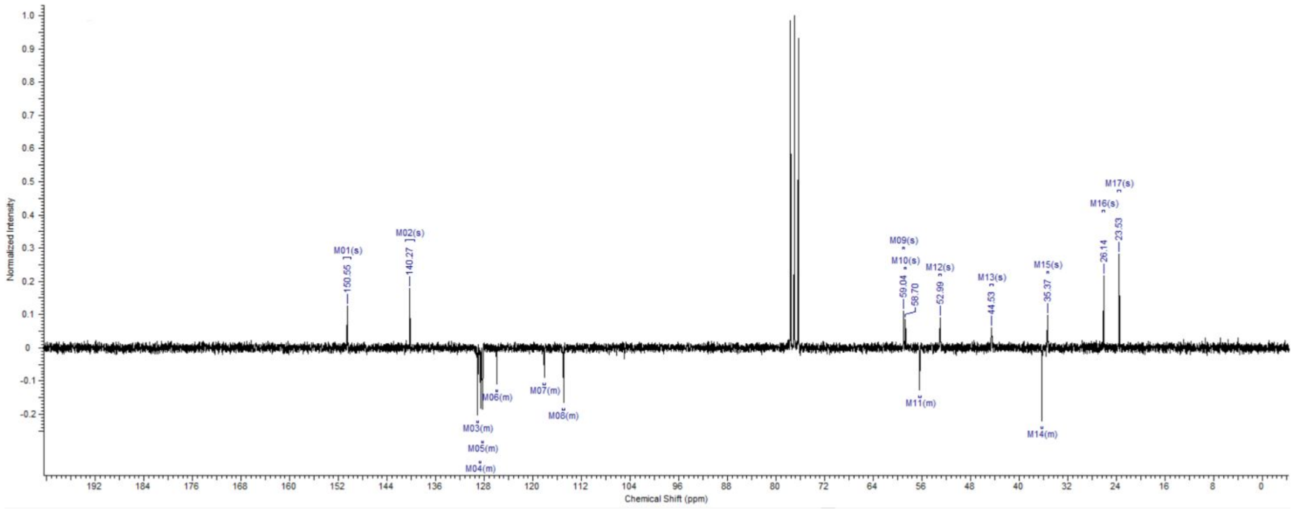

6-Benzyl-1-phenethyloctahydro-1*H*-pyrrolo[3,4-*b*]pyridine (**AB9**, **8c**)

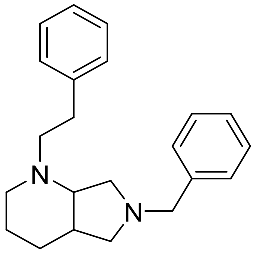

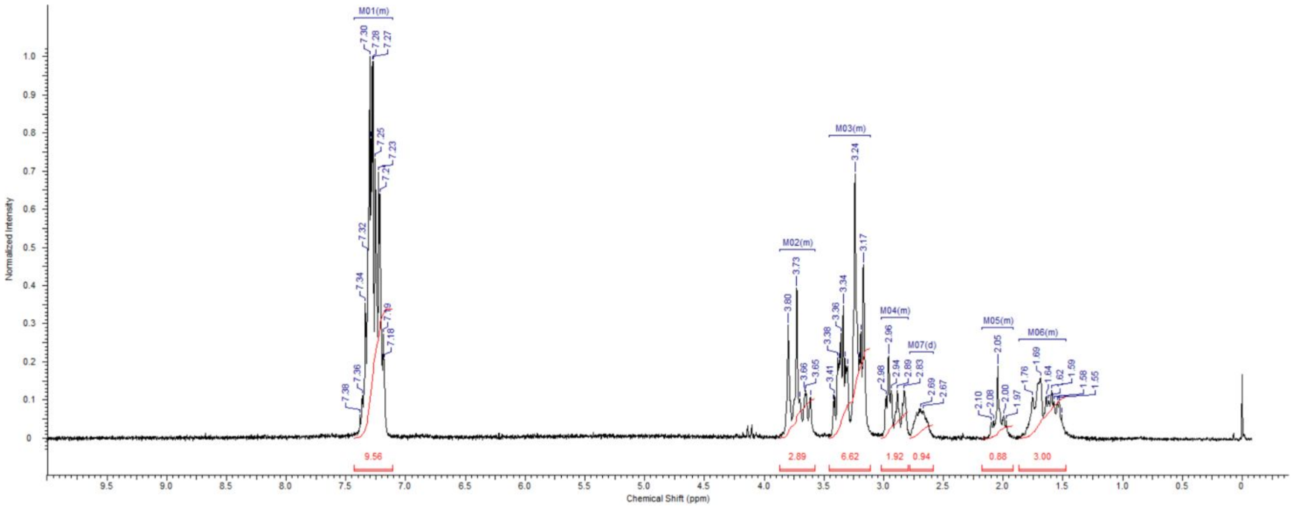

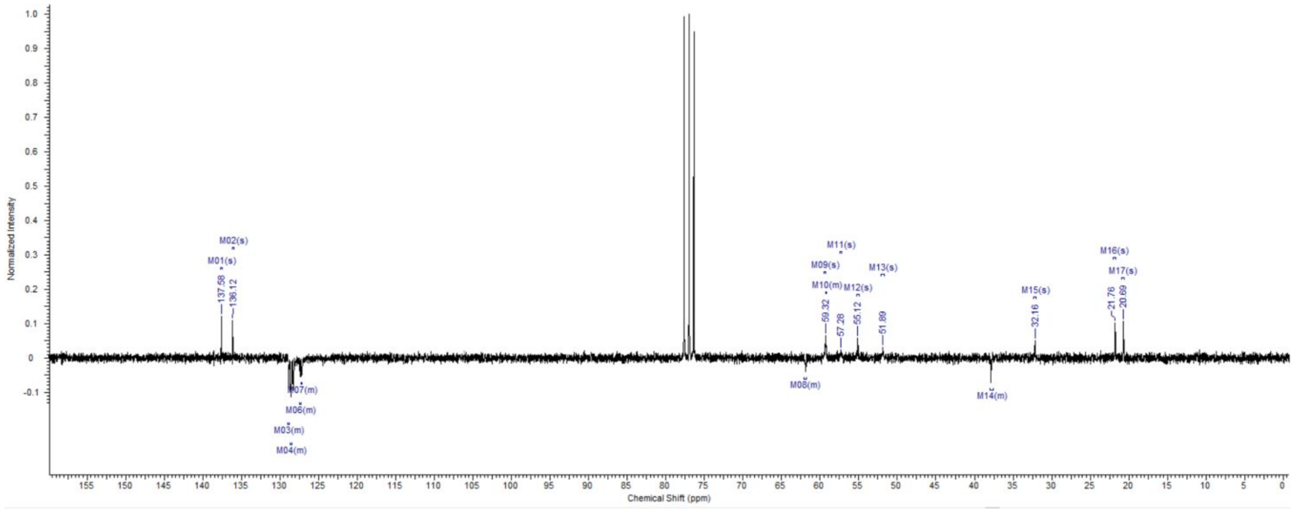

1,6-Diphenethyloctahydro-1*H*-pyrrolo[3,4-*b*]pyridine (**AB7**, **8d**)

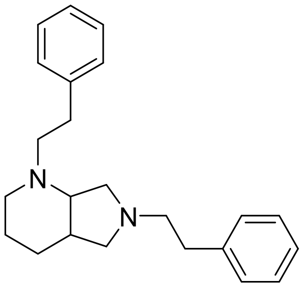

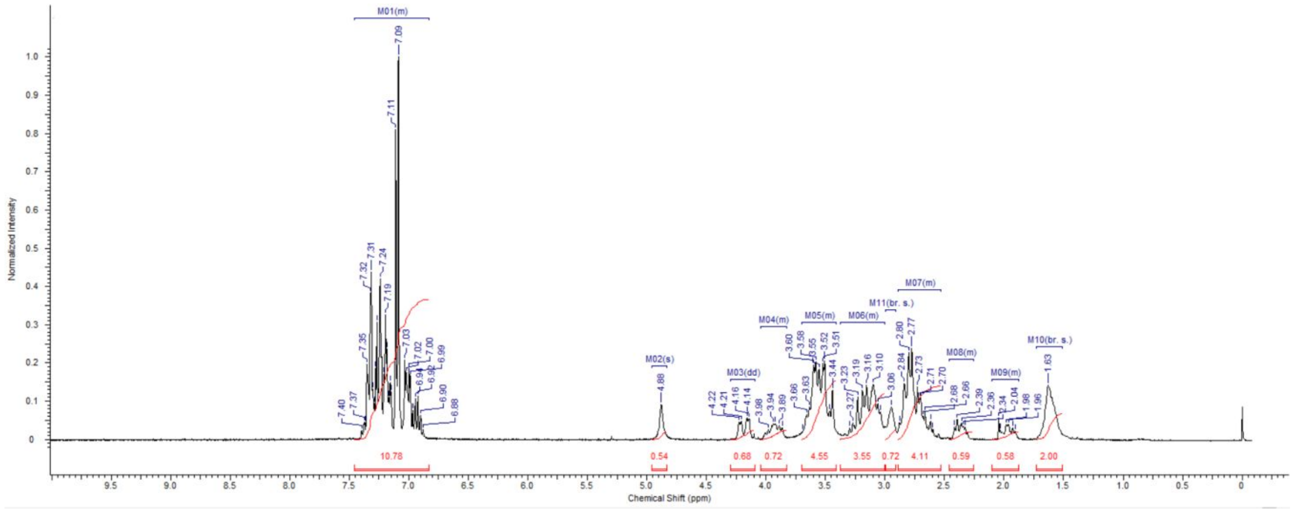

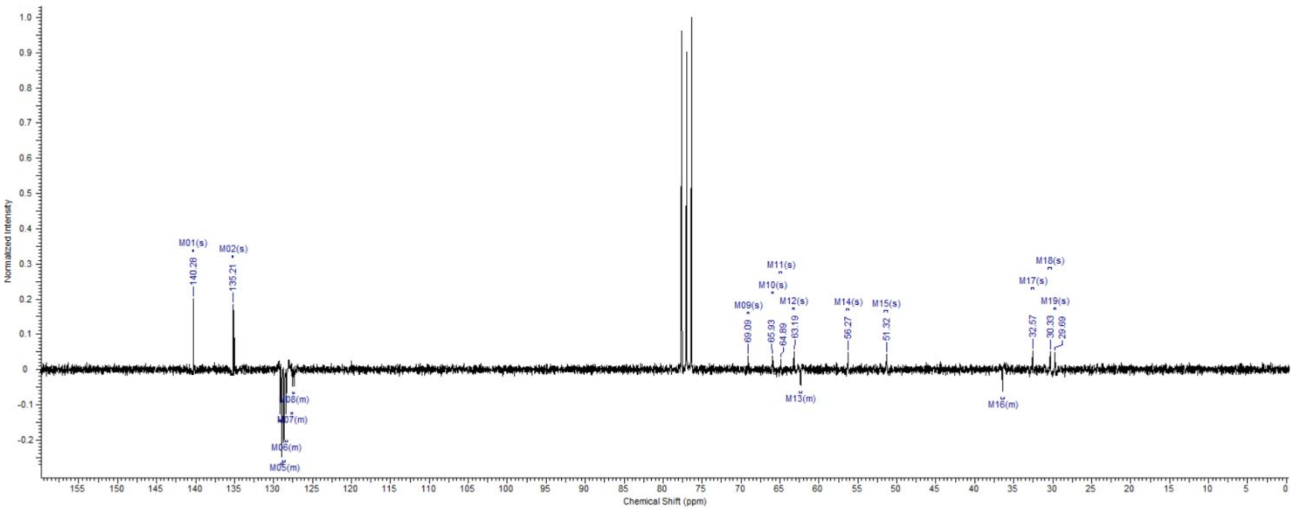

6-Phenyl-1-(3-phenylpropyl)octahydro-1*H*-pyrrolo[3,4-*b*]pyridine (**AB14**, **8e**)

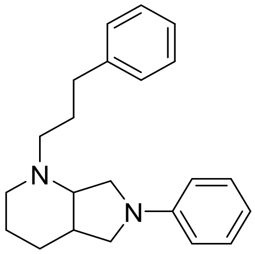

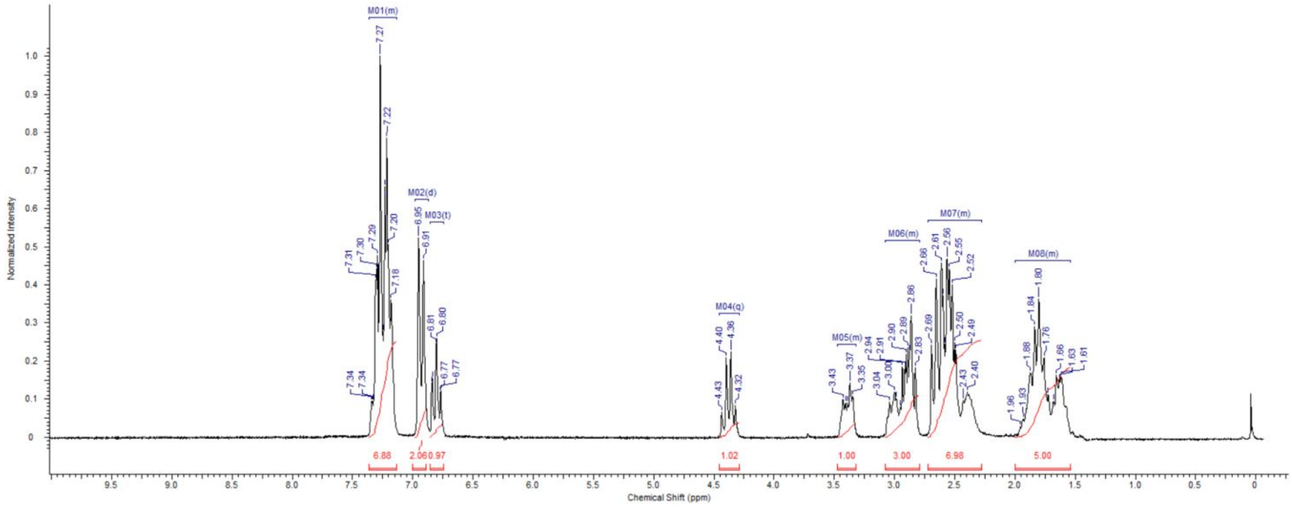

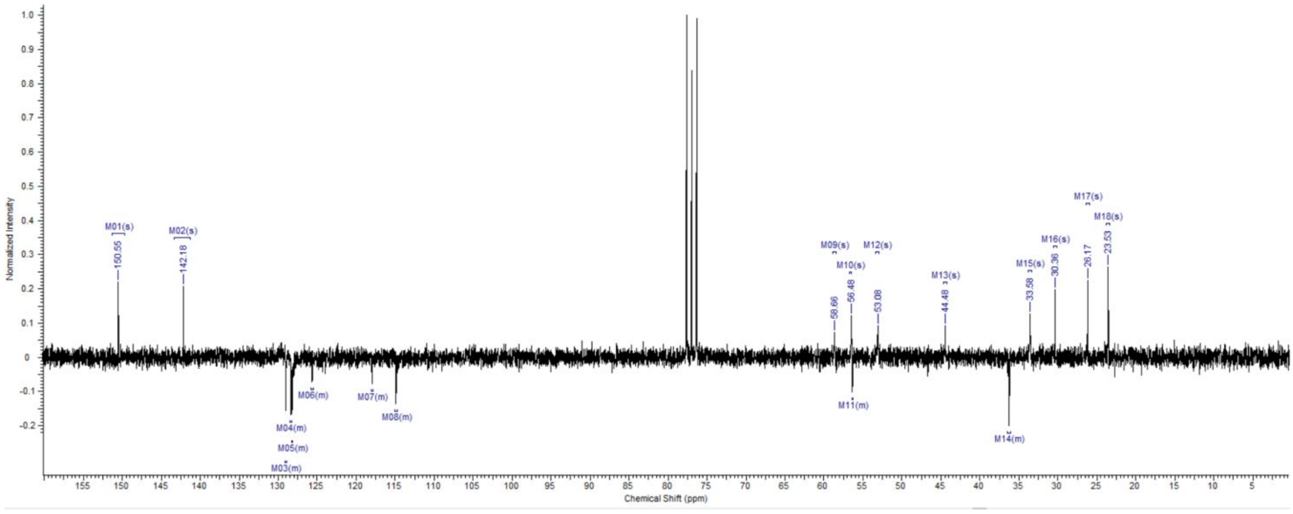

6-Benzyl-1-(3-phenylpropyl)octahydro-1*H*-pyrrolo[3,4-*b*]pyridine (**AB10**, **8f**)

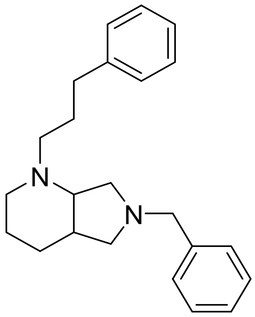

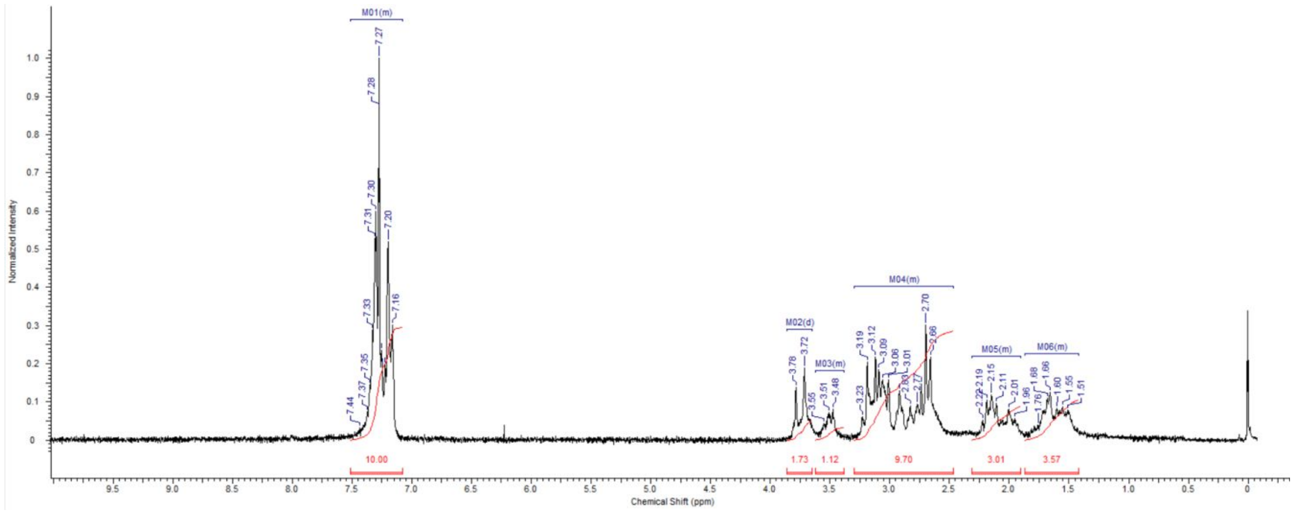

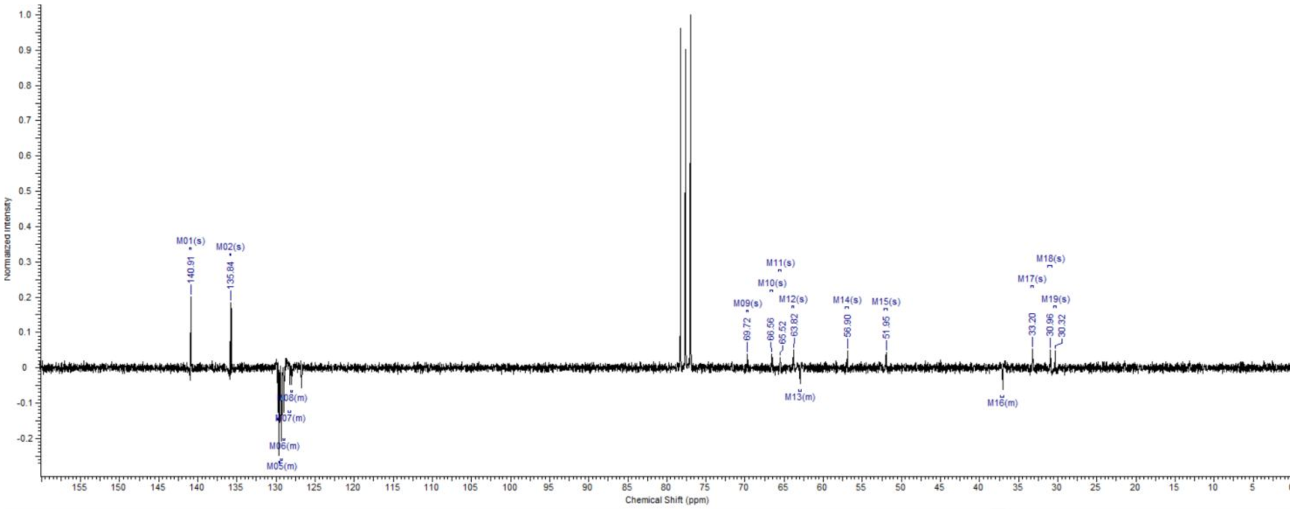

6-Phenethyl-1-(3-phenylpropyl)octahydro-1*H*-pyrrolo[3,4-*b*]pyridine (**AB8**, **8g**)

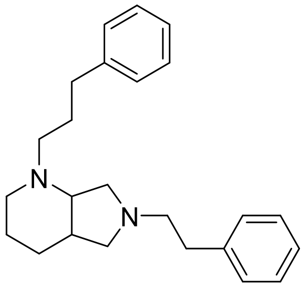

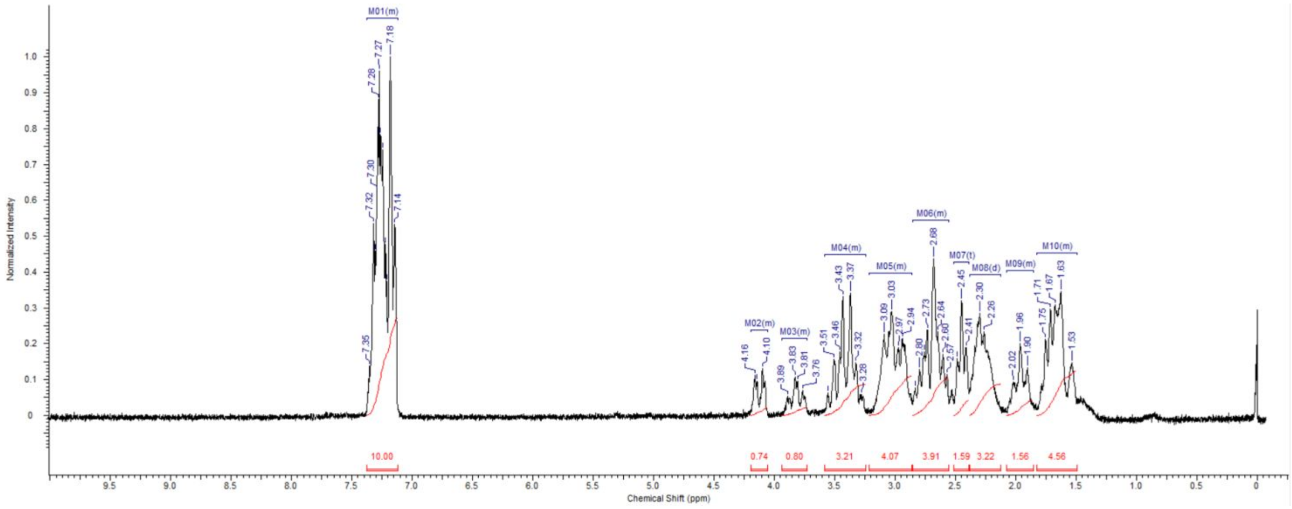

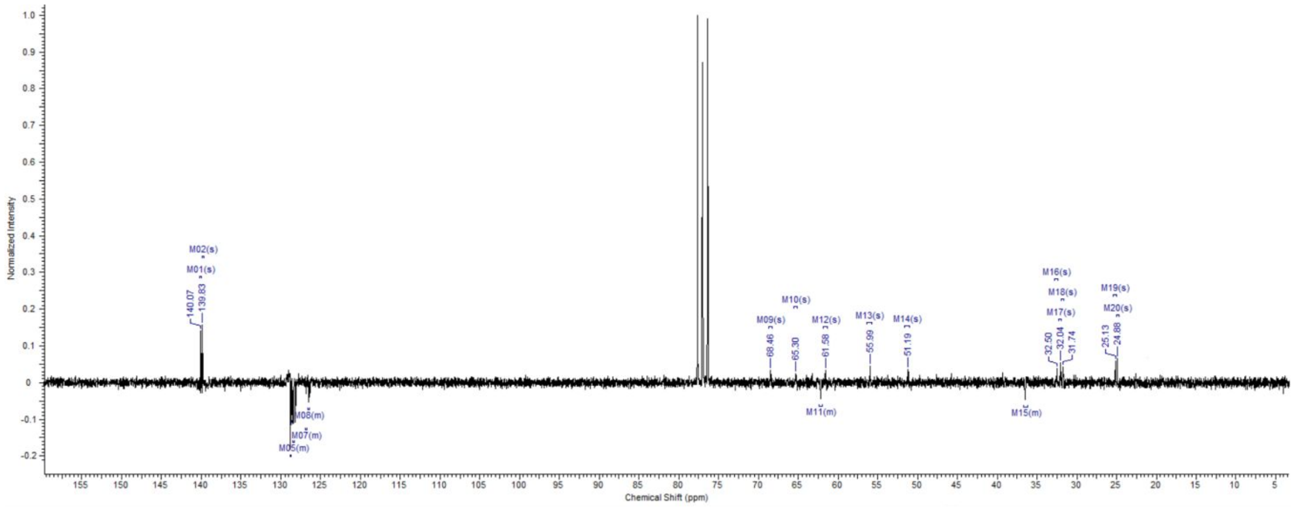

Supplement: Supplementary file 1 — cn3c00074_si_001.pdf [file cn3c00074_si_001.pdf]
